# Identification and characterization of drought-responsive CC-type glutaredoxins from cassava cultivars reveals their involvement in ABA signalling

**DOI:** 10.1186/s12870-018-1528-6

**Published:** 2018-12-04

**Authors:** Meng-Bin Ruan, Yi-Ling Yang, Kai-Mian Li, Xin Guo, Bin Wang, Xiao-Ling Yu, Ming Peng

**Affiliations:** 1 Institute of Tropical Bioscience and Biotechnology, Chinese Academy of Tropical Agricultural Sciences, Haikou, 571101 China; 2 Guangdong Provincial Key Laboratory of Crop Genetic Improvement, Crops Research Institute, Guangdong Academy of Agricultural Sciences, Guangzhou, 510640 China; 3 Tropical Crops Genetic Resources Institute, Chinese Academy of Tropical Agricultural Science, Danzhou, 571701 China; 4 Huazhong Agricultural University, Wuhan, 430070 China; 5 Key Laboratory of Biology and Genetic Resources of Torpical Crops, Ministry of Agriculture, Haikou, 571101 China

**Keywords:** Cassava (*Manihot esculenta*), Drought response, ABA, CC-type glutaredoxin, Transgenic *Arabidopsis*, TGA factors

## Abstract

**Background:**

CC-type glutaredoxins (GRXs) are plant-specific glutaredoxin, play regulatory roles in response of biotic and abiotic stress. However, it is not clear whether the CC-type GRXs are involve in drought response in cassava (*Manihot esculenta*), an important tropical tuber root crop.

**Results:**

Herein, genome-wide analysis identified 18 CC-type GRXs in the cassava genome, of which six (namely *MeGRXC3, C4, C7, C14, C15*, and *C18*) were induced by drought stress in leaves of two cassava cultivars Argentina 7 (Arg7) and South China 124 (SC124). Exogenous abscisic acid (ABA) application induced the expression of all the six CC-type GRXs in leaves of both Arg7 and SC124 plants. Overexpression of *MeGRXC15* in *Arabidopsis* (Col-0) increases tolerance of ABA on the sealed agar plates, but results in drought hypersensitivity in soil-grown plants. The results of microarray assays show that *MeGRXC15* overexpression affected the expression of a set of transcription factors which involve in stress response, ABA, and JA/ET signalling pathway. The results of protein interaction analysis show that MeGRXC15 can interact with TGA5 from *Arabidopsis* and MeTGA074 from cassava.

**Conclusions:**

CC-type glutaredoxins play regulatory roles in cassava response to drought possibly through ABA signalling pathway.

**Electronic supplementary material:**

The online version of this article (10.1186/s12870-018-1528-6) contains supplementary material, which is available to authorized users.

## Background

As a tropical crop, cassava (*Manihot esculenta*) evolved different responses to drought stress, such as quick stomata closure, reduction of photosynthetic proteins levels and photosynthetic capacity, induction of senescence in older leaves, and size reduction of leave epidermal cells [1, 2]. The cassava cultivar Argentina 7 (Arg7) display faster senescence in older leaves than the cassava cultivar South China 124 (SC124) [2]. Senescence in cassava is partly controlled by reactive oxygen species (ROS) and ethylene (ET) signaling [3]. Increasing ROS scavenging ability in cassava delays leaf senescence under drought stress [3–5]. It is therefore necessary to analyze genes involved in these pathways for a deeper functional characterization.

Glutaredoxin (GRX) is one of the most important protein modification system in plants [6]. The glutathione/GRX (GSH/GRX) system is essential for redox homeostasis and ROS signalling in plant cells [7]. GRX target proteins are involved in all aspects of plant growth, including basal metabolism, iron/sulfur cluster formation, development, adaptation to the environment, and stress responses [7]. GRX are in particular studied for their involvement in oxidative stress responses [7–9]. GRXs are classified in five subgroups, among which CC-type GRXs are a plant-specific subgroup, also known as the ROXY family in *Arabidopsis* [10, 11]. CC-type GRXs likely evolved from the CPYC subgroup and expanded during land plant evolution [11]. There are only two CC-type GRXs in the basal land plant *Physcomitrella,* but between 15 and 24 members in land plants such as rice, *Arabidopsis, Vitis* and *Populus* [11]. However, the number of CC-type GRXs in cassava remains unclear.

CC-type GRXs are characterized by the presence of a redox site CC*(C/S/G) as well as their disulfide reductase activity that uses glutathione as cofactor [12]. The first CC-type GRX has been identified as a regulator of petal development [10]. However, CC-type GRXs are also involved in jasmonic acid (JA) / ET mediated biotic stress responses through the interaction with TGA transcription factors in *Arabidopsis* [13–15]. Moreover, a CC-type GRX GRXS13 is critical in limiting basal and photo-oxidative stress-induced ROS production [16]. Thus, CC-type GRXs may play a key role in the crosstalk between ROS and ethylene. CC-type GRX members are also involved in organ development and biotic stress responses in other plants [13, 17–21]. Since ROS and ethylene signal transduction pathways are involved in cassava drought responses [3], CC-type GRXs might play regulatory roles in these pathways in cassava.

During evolution, CC-type GRXs might have gained new functions in higher land plants [6, 11, 13]. Although several CC-type GRXs have been characterized in *Arabidopsis* and rice [13, 21], no previous work has profiled them in cassava. Moreover, protein interactions and enzymatic activities during abiotic stress responses in cassava are equally important [2], but currently not well understood. With the recent release and annotation of the cassava genome [22, 23], it is now possible to identify drought responsive CC-type GRX genes and to better characterize their functions and interactors during drought response.

Based on our previously reported transcriptomic data of cassava cultivars [24], we identified six CC-type GRXs (*MeGRXC3, C4, C7, C14, C15,* and *C18*) that responded to drought using qPCR analysis in cassava leaves. Expression of the six drought-responsive CC-type GRXs is also induced in cassava leaves by application of exogenous ABA. Our results showed that CC-type GRXs may function as a component in drought stress in an ABA-dependent pathway in both Arg7 and SC124 plants. Furthermore, we found that *MeGRXC15* is specifically expressed by exposure to drought in different tissues including leaf, petiole, and abscission zone. Overexpression of *MeGRXC15* in *Arabidopsis* induces insensitivity to ABA on sealed agar plates, and confers drought susceptibility in soil-grown conditions. In addition, gene expression analysis reveals that *MeGRXC15* overexpression in *Arabidopsis* altered the expression of a set of genes which involve in multiple stress responses, ABA, and JA/ET signalling pathways. Protein-protein interaction analysis reveals that MeGRXC15 could interact with TGA transcription factor from both *Arabidopsis* and cassava. Together, our study could increase the understanding of cassava gene regulation under the condition of drought stress and expand the knowledge of land plant specific CC-type GRXs.

## Results

### Phylogenetic and protein sequence analysis of cassava CC-type GRXs

**Fig. 1:**
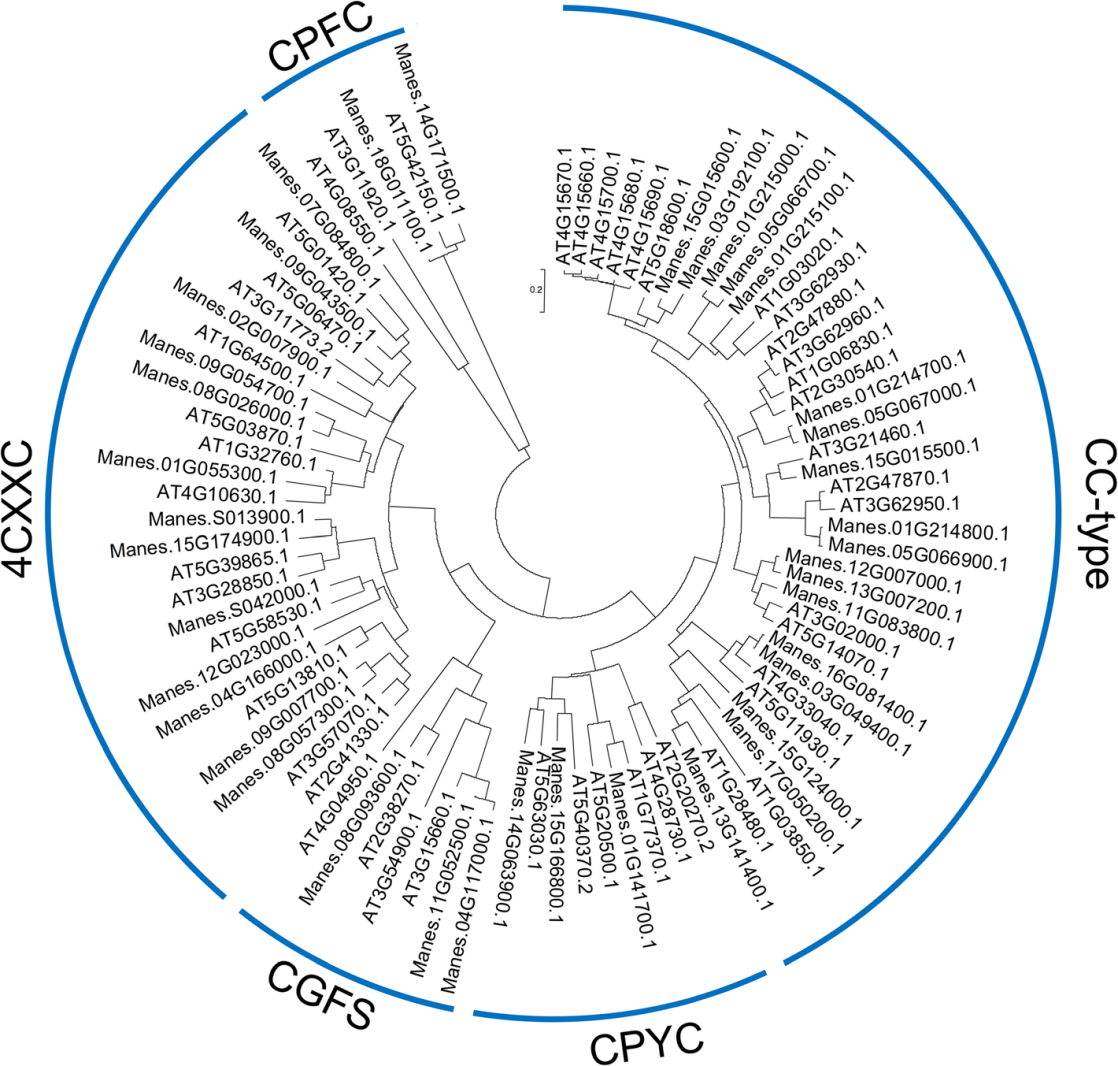
Phylogenetic tree of glutaredoxins (GRXs) from cassava and *Arabidopsis*. Members of GRXs were classified by their redox activate site

**Fig. 2:**
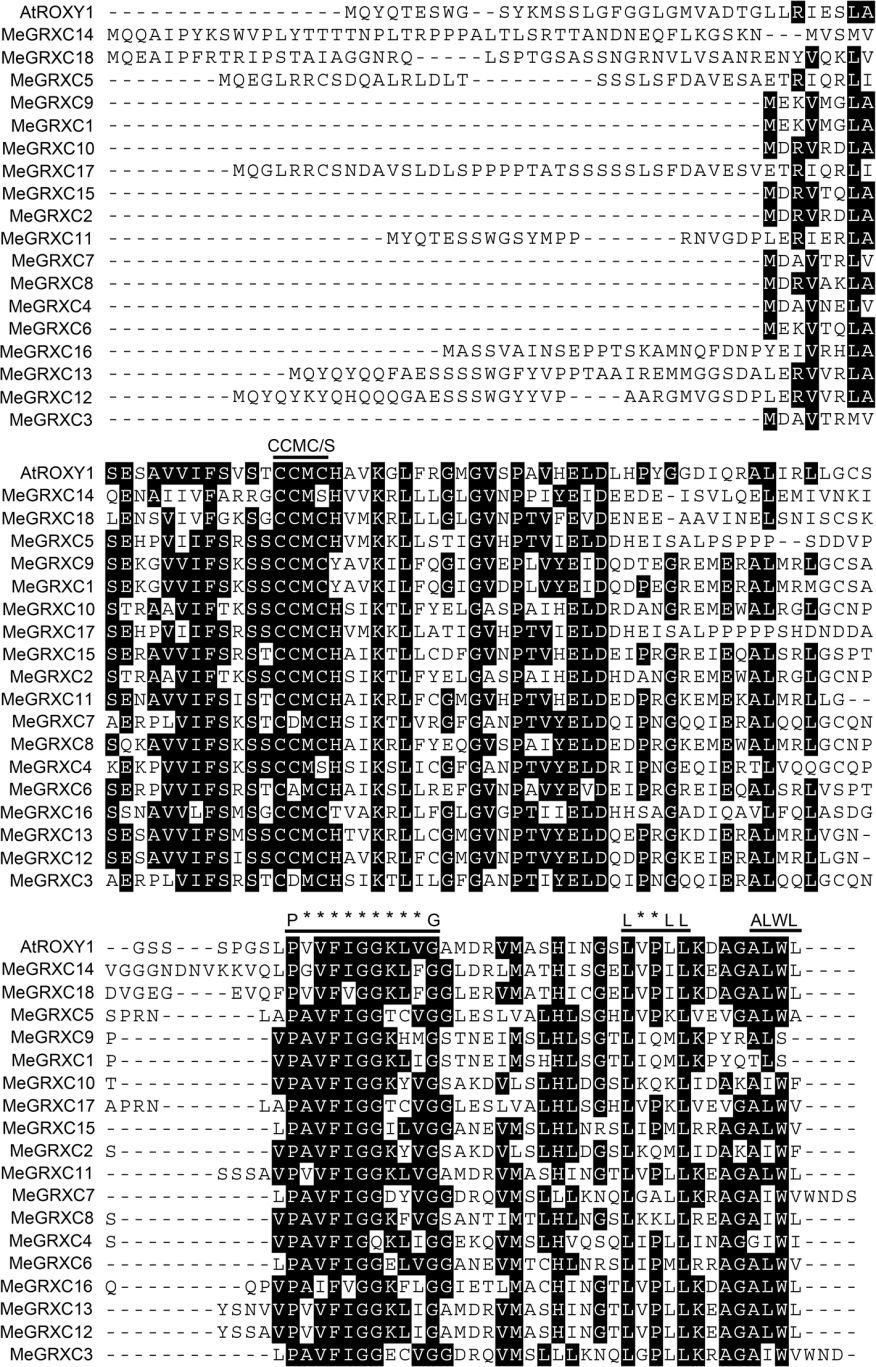
Protein sequences alignment of cassava CC-type GRXs. Black boxes indicate conserved identify positions. The letters above the sequence indicate motif name

We predicted a total of 39 putative GRX proteins in the cassava genome using the *Arabidopsis* ROXYs in a BLAST search against the genome of the cassava cultivar AM560 (https://phytozome.jgi.doe.gov/pz/portal.html, *Manihot esculenta v6.1*). To understand the relationship between GRX proteins in cassava and *Arabidopsis,* we built a neighbor-joining phylogenetic tree using MEGA5.0 on the basis of the protein sequences in Additional file 1: Table S1. The results show that many cassava GRXs are highly similar to their *Arabidopsis* counterparts (Fig. 1). We found that the CC-type subgroup had the most members among five GRX subgroups in cassava. Our analysis predicted 18 full-length CC-type GRX members of cassava (Table 1), less than the 21 of *Arabidopsis* [25]. Cassava CC-type GRX genes are represented on nine chromosomes (Table 1). 16 of cassava CC-type GRXs share an ALWL motif at the C-terminus and extend to A(G)L(I)WL(A/F/V/I) (Fig. 2, Table 1). However, two members, MeGRXC1 and MeGRXC9, are not present ALWL motif at the C-terminus (Fig. 2, Table 1). CC-type GRXs have a distinctive conserved CC(M/L)(C/S) redox site motif in *Arabidopsis*, whereas this motif extends to C(C/G/F/Y/P)(M/L)(C/S/I/A) in rice [6, 11, 13, 25]. Most cassava CC-type GRXs shares a distinctive CCM(C/S) redox site (Fig. 2, Table 1). However, CDMC is extended in two CC-type GRXs (MeGRXC3 and MeGRXC7) and CAMC is extended in MeGRXC6 in cassava (Fig. 2, Table 1).

**Table 1:** CC-type GRXs in cassava genome

JGI identifier V4.1	JGI identifier V6.1	Chromosome location	Gene Symbol	Redox Site	ALWL-motif	AA
cassava4.1_019956m	Manes.01G214700.1	Chr01:30392540..30393324	MeGRXC1	CCMC	–	102
cassava4.1_024597m	Manes.01G214800.1	Chr01:30394902..30395210	MeGRXC2	CCMC	AIWF	103
cassava4.1_033785m	Manes.01G215000.1	Chr01:30421882..30422775	MeGRXC3	CDMC	AIWV	105
cassava4.1_027058m	Manes.01G215100.1	Chr01:30426232..30427641	MeGRXC4	CCMS	GIWI	103
cassava4.1_018918m	Manes.03G049400.1	Chr03:4265637..4266041	MeGRXC5	CCMC	ALWA	135
cassava4.1_027873m	Manes.03G192100.1	Chr03: 27589330..27589919	MeGRXC6	CAMC	ALWV	102
cassava4.1_025892m	Manes.05G066700.1	Chr05:5107836..5108531	MeGRXC7	CDMC	AIWV	106
cassava4.1_021286m	Manes.05G066900.1	Chr05:5120724..5122777	MeGRXC8	CCMC	AIWL	103
cassava4.1_019954m	Manes.05G067000.1	Chr05:5123236..5123953	MeGRXC9	CCMC	–	102
cassava4.1_024608m	Manes.11G083800.1	Chr11:11464621..11464992	MeGRXC10	CCMC	AIWF	124
cassava4.1_032936m	Manes.12G007000.1	Chr12:652588..653004	MeGRXC11	CCMC	ALWL	139
cassava4.1_032796m	Manes.13G007200.1	Chr13:771539..771955	MeGRXC12	CCMC	ALWL	139
cassava4.1_018177m	Manes.13G141400.1	Chr13:26980309..26981228	MeGRXC13	CCMS	ALWL	157
cassava4.1_026496m	Manes.15G015500.1	Chr15:1268904..1269746	MeGRXC14	CCMC	ALWL	123
cassava4.1_024232m	Manes.15G015600.1	Chr15:1265181..1265486	MeGRXC15	CCMC	ALWV	102
cassava4.1_028408m	Manes.15G124000.1	Chr15:9399566..9399949	MeGRXC16	CCMC	ALWL	128
cassava4.1_024091m	Manes.16G081400.1	Chr16:23848065..23848499	MeGRXC17	CCMC	ALWV	145
cassava4.1_018360m	Manes.17G050200.1	Chr17:18791502..18792218	MeGRXC18	CCMC	ALWL	151

### Identification of drought-inducible CC-type GRX genes in cassava cultivar Arg7 and SC124

**Fig. 3:**
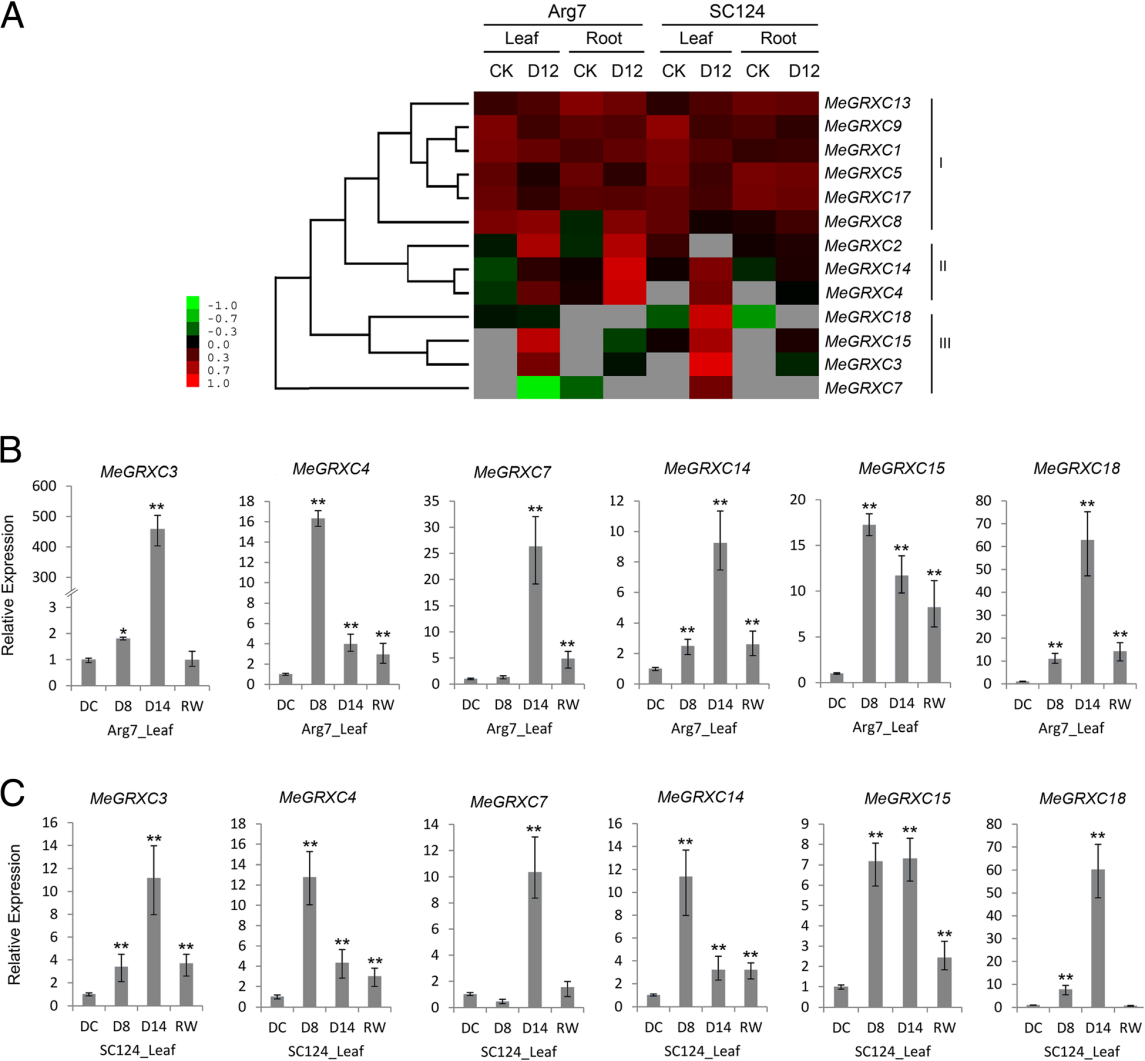
Expression analysis of drought-responsive CC-type GRX genes in cassava cultivar Arg7 and SC124. **a** Heat map represent expression of CC-type GRXs in drought stressed cassava Arg7 and SC124. **b** and (**c**) qPCR analysis of *MeGRXC3, C4, C7, C14, C15*, and *C18* in drought stressed leaves of cassava Arg7and SC124. DC: control; D8: eight days after water withholding; D14: 14 days after water withholding; RW: rewatered D14 plants 24 h after rehydration. Expression levels of the six CC-type GRXs were normalized against DC. Biological triplicates were averaged and significance of differences between treatments and control were analyzed using the *Student’s t-test* (**, *p* ≤ 0.01). Bars represent the mean ± standard error

Previous studies used RNA-seq datasets to examine genes responsive to drought resistance in cassava [26–30]. RNA-seq datasets are available at NCBI and the accession numbers are listed in Additional file 2: Table S2. To investigate the expression of CC-type GRXs in response to drought in cassava, we used our previously reported transcriptomic data from cassava cultivar Arg7 and SC124 (Additional file 2: Table S3). Hierarchical expression clustering (FPKM) result shows that the CC-type GRX expression patterns in response to drought in cassava cultivars Arg7 and SC124 grouped in three clusters (Fig. 3a). Cluster II include CC-type GRXs induced by drought in both leaf and root. Cluster III include CC-type GRXs induced by drought only in leaves. To detail the expression of CC-type GRXs in the drought response in cassava leaves, we performed a qPCR analysis to investigate the expression changes of genes in cluster II and III under drought and re-water treatments. For this analysis, we selected six drought-inducible CC-type GRXs (*MeGRXC3, C4, C7, C14, C15,* and *C18*) from cluster II and III. We collected leaves from plants of two cassava cultivars under drought stress for eight (D8) or 14 days (D14), and rewatered D14 plants 24 h after rehydration (RW). We used leaves from well-watered cassava plants as control (DC). The qPCR results show that drought stress up-regulated the expression of all six CC-type *GRX*s in both Arg7 and SC124 leaves (Fig. 3b, c). The expression of *MeGRXC3, C7* and *C18* was the highest in D14 plants of both two cultivars. In contrast, the expression of *MeGRXC4* and *C15* was the highest in D8 plants of both two cultivars (Fig. 3b, c). Additionally, the expression of *MeGRXC14* was the highest in D14 plants of Arg7 (Fig. 3b), while it was the highest in D8 plants of SC124 (Fig. 3c).

### Cassava drought-responsive CC-type GRXs are induced by ABA in leaves

**Fig. 4:**
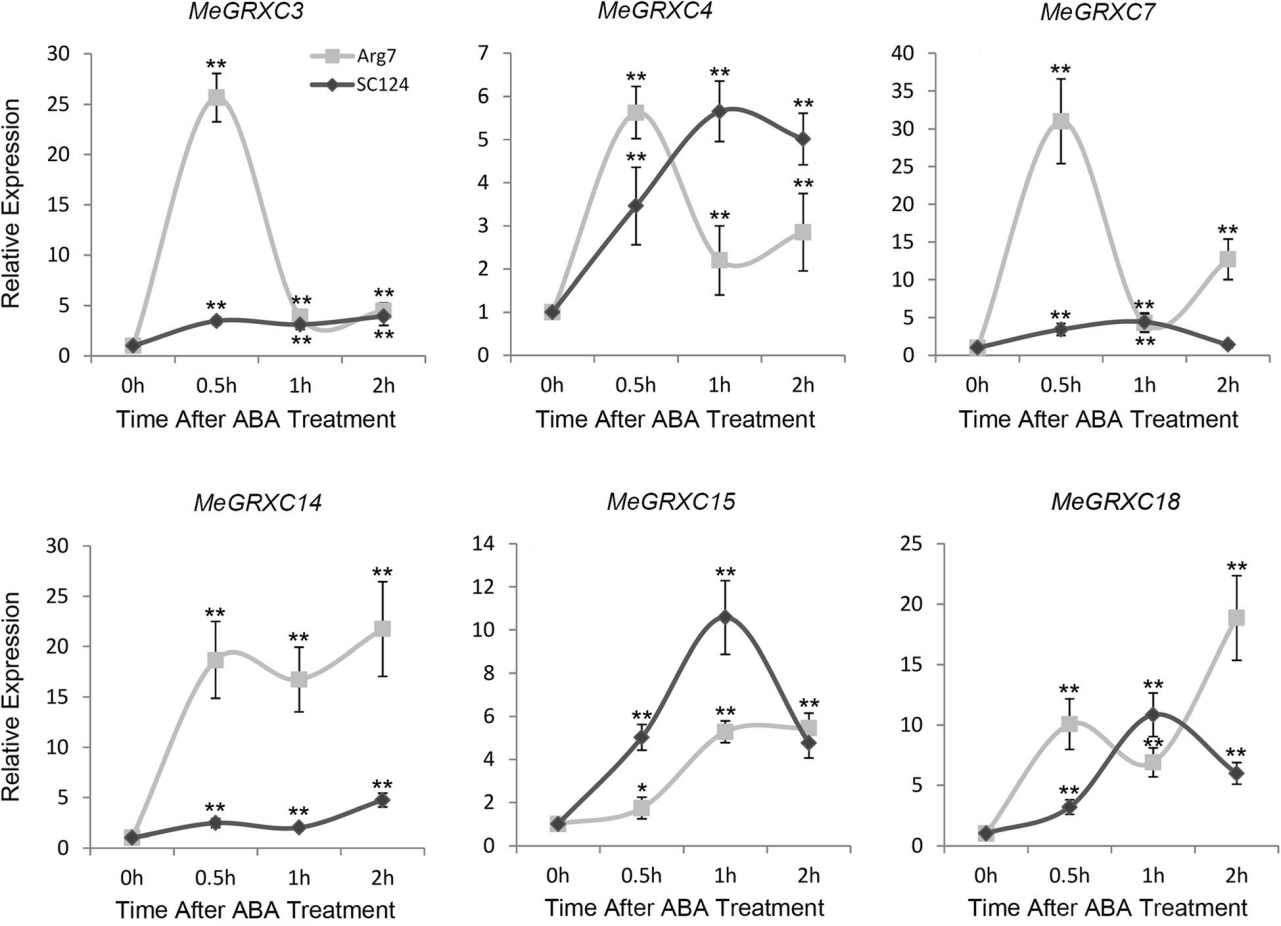
Effects of ABA on expression of the six drought-responsive CC-type GRXs in cassava cultivar Arg7 and SC124 leaves. Expression levels of the six CC-type GRXs were normalized against control. Biological triplicates were averaged and significance of differences between treatments and control were analyzed using the *Student’s t-test* (**, *p* ≤ 0.01; *, 0.01 < *p* ≤ 0.05). Bars represent the mean ± standard error

Numerous drought-responsive genes were described as ABA-inducible [31]. We performed qPCR analysis to investigate whether our drought-responsive CC-type GRXs are regulated by ABA in cassava leaves. We found that ABA application up-regulated the expression of six drought-responsive CC-type GRXs in leaves of Arg7 and SC124 (Fig. 4). The *MeGRXC3, C14*, and *C15* showed similar ABA-induced expression patterns between Arg7 and SC124 (Fig. 4). This indicates that CC-type GRXs involving in ABA-dependent pathway during cassava response to drought.

### Cassava drought-responsive CC-type GRXs are localized in the nucleus and cytoplasm

**Fig. 5:**
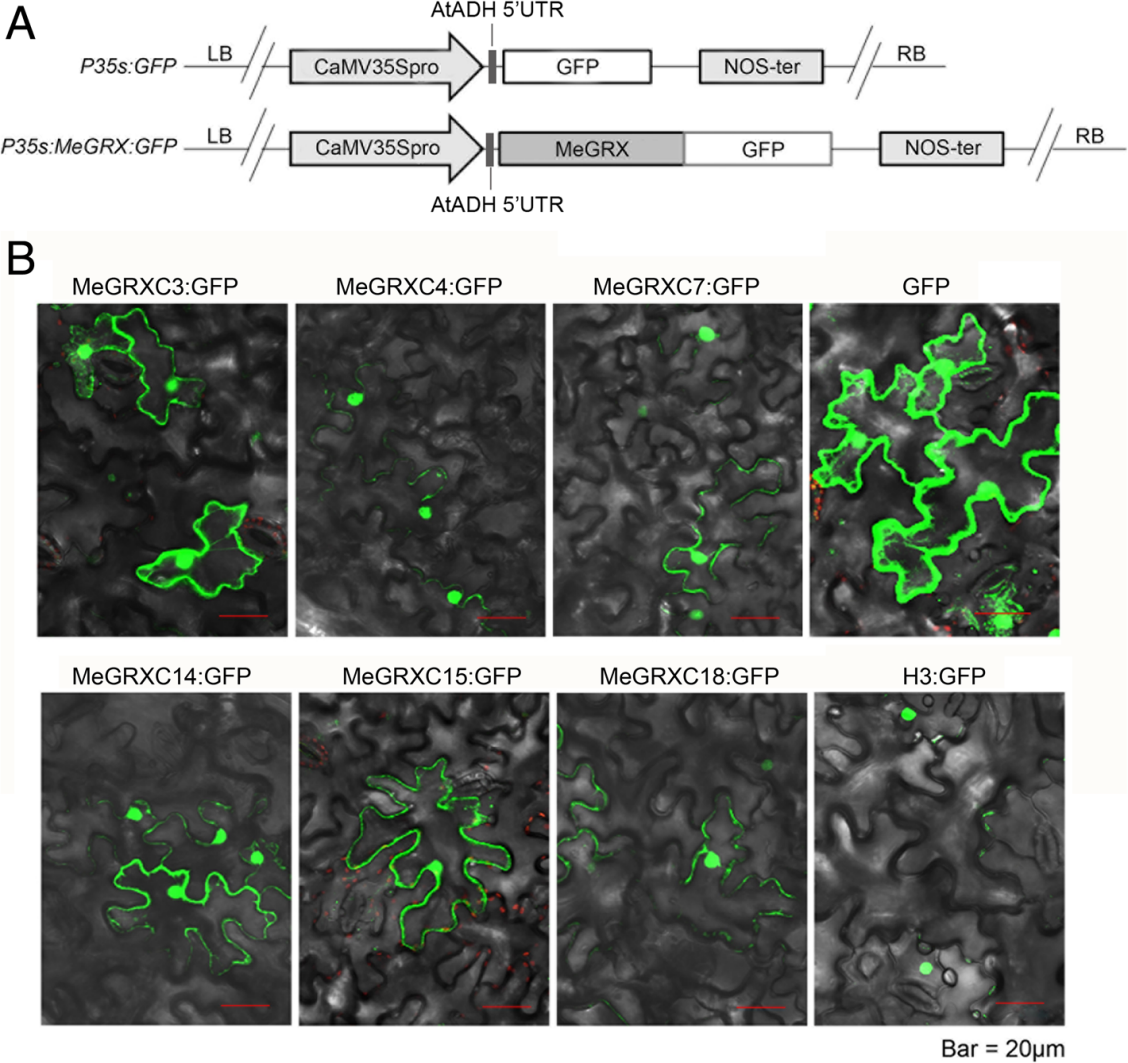
Protein localization analysis of the six drought-responsive CC-type GRXs. **a** Schematic diagram represent the design of *35S:MeGRX:GFP* constructs. **b** Subcellular localization of MeGRX:GFP fusion proteins transiently expressed in *N. benthamiana* leaves. GFP was used as expression control, H3:GFP (H3, *histone 3*) was used as nuclear expression control

Most *Arabidopsis* CC-type GRX proteins localize in the cytosol or in the nucleus [10, 25, 32]. We respectively tagged the cDNA of *MeGRXC3, C4, C7, C14, C15,* and *C18* with *GFP* at the C-terminus to analyze their cellular localization (Fig. 5a). We imaged the MeGRX:GFP fusion proteins in transiently transformed *N.benthamiana* leaf epidermis, detecting fluorescence in both the cytosol and the nucleus (Fig. 5b). The nuclear localization of MeGRX:GFP fusion proteins indicates that these drought-responsive CC-type GRXs may play roles in the nucleus during drought responses.

### *MeGRXC15* is tissue specifically induced by drought in cassava cultivars

**Fig. 6:**
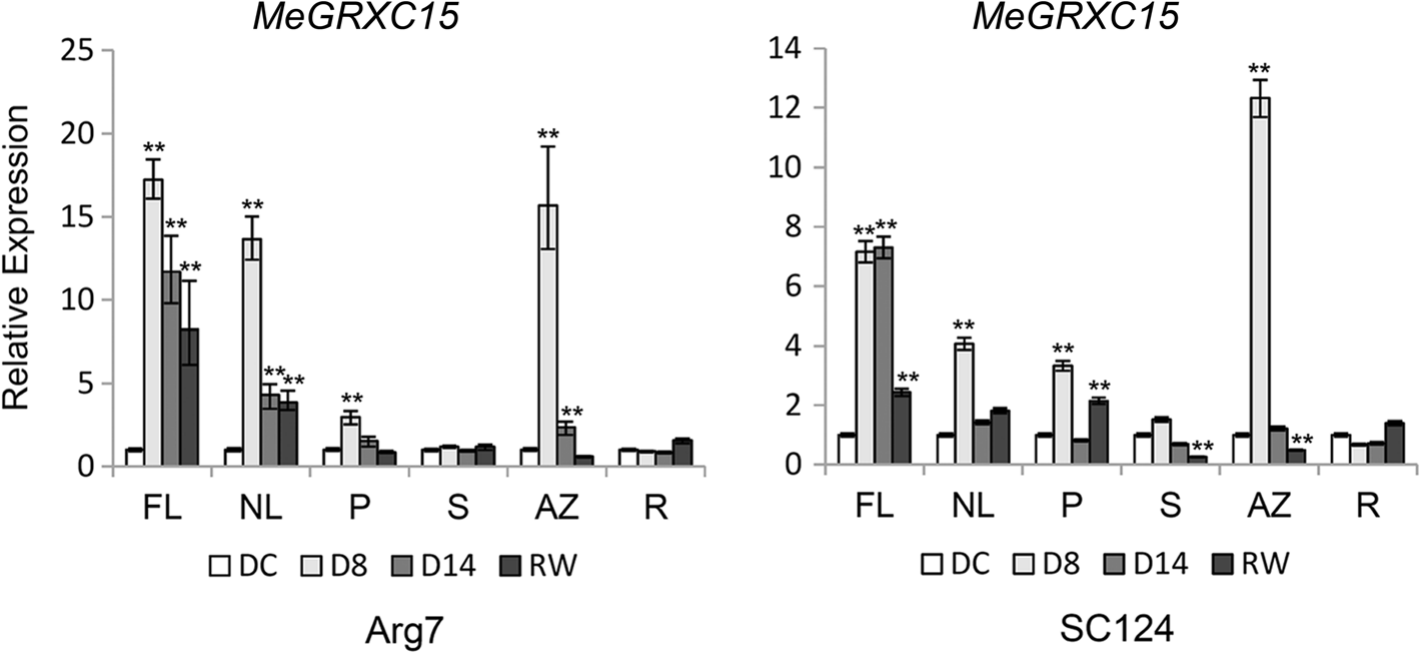
Expression analyses of *MeGRXC15* in different tissues from drought stressed cassava cultivar Arg7 and SC124. FL, functional leaf; NL, new leaf; P, petiole; S, stem; AZ, abscission zone; R, root; DC: control; D8: eight days after water withholding; D14: 14 days after water withholding; RW: rewatered D14 plants 24 h after rehydration. Expression levels of the *MeGRXC15* were normalized against DC. Biological triplicates were averaged and significance of differences between treatments and control were analyzed using the *Student’s t-test* (**, *p* ≤ 0.01). Bars represent the mean ± standard error

For the *MeGRXC15* shows similar expression pattern in leaves of both cassava cultivars Arg7 and SC124 under drought and ABA treatments (Figs. 3 and 4), this gene was selected for further investigation. Regulation of temporal and spatial expression patterns of specific stress-responsive is an important part of plant drought responses [33]. We performed qPCR analysis to examine the expression pattern of *MeGRXC15* in different tissues of drought stressed cassava. We found that expression of *MeGRXC15* was dramatically up-regulated by drought in functional leaf (FL), new leaf (NL), petiole (P), and abscission zone (AZ) of both cassava cultivars Arg7 and SC124 (Fig. 6). Abscission zone initiation in drought stressed cassava is based on reactive oxygen species (ROS) and ethylene (ET) signal transduction [3]. The *MeGRXC15* shown higher induced expression level in AZ at D8 suggesting its potential roles in ROS or ET signal transduction pathway.

### *MeGRXC15* confers seedling development insensitive to ABA and drought hypersensitivity in soil-grown plants

**Fig. 7:**
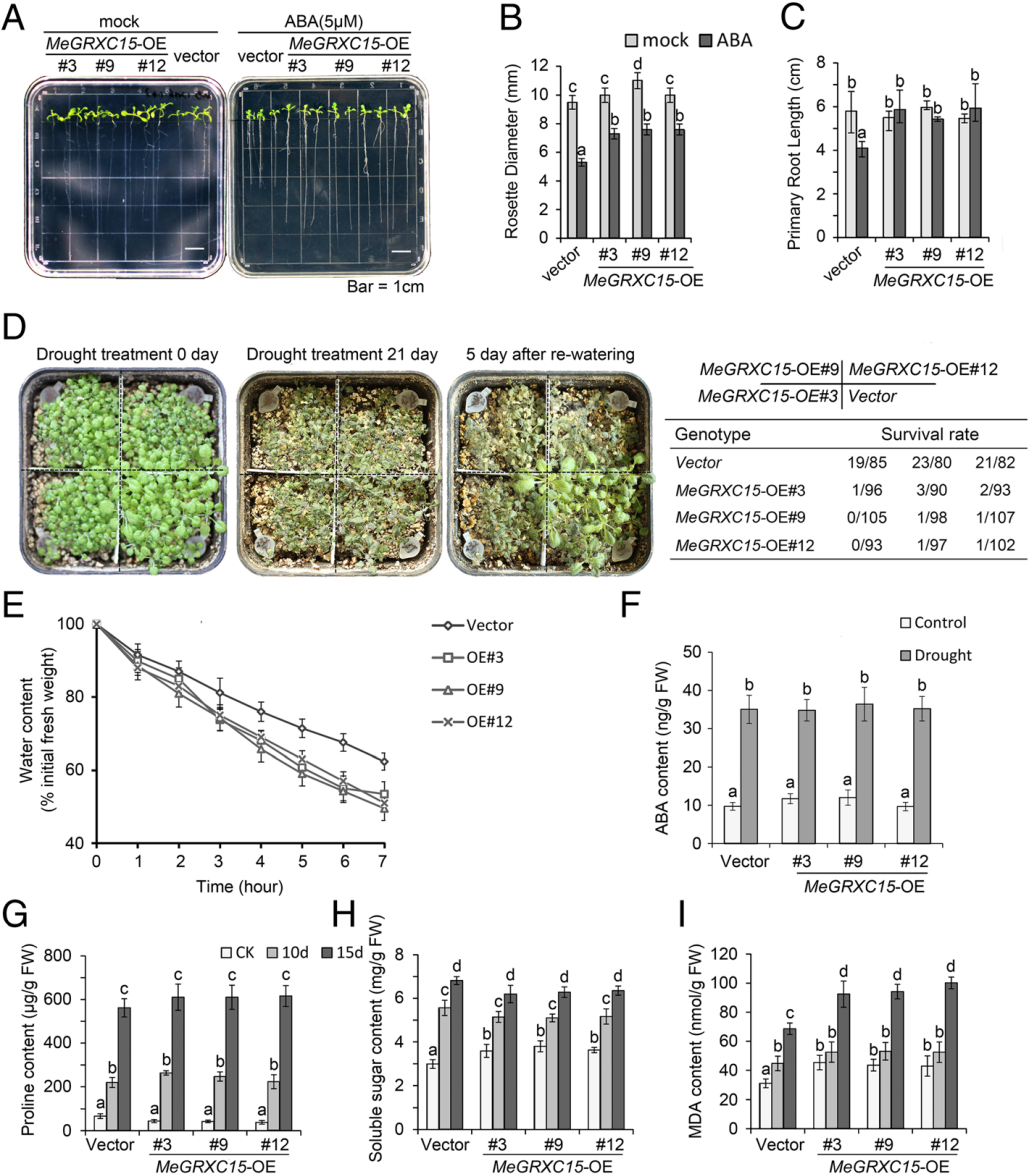
ABA and drought tolerance analyses of *MeGRXC15-OE* transgenic *Arabidopsis.* **a** Post-germinated seedlings development of transgenic plants on MS medium supplemented with 0 (mock) and 5 μM ABA, respectively. The plants that contained empty vector (*pG1300*) were used as control. **b** and (**c**) Rosette diameter and primary root length of transgenic *Arabidopsis* under ABA treatment. **d** Drought responses of transgenic plants. Survival rates were calculated from three independent experiments. **e** Water loss rate analysis of transgenic *Arabidopsis*. **f** Endogenous ABA content in transgenic *Arabidopsis* under normal and drought conditions. Proline content (**g**), soluble sugar (**h**), and MDA content (**i**) in transgenic plants under drought treatment. Biological triplicates were averaged and significance of difference between treatments and control was analyzed using the Duncan’s multiple range tests. Different letters represent a significant difference at *p* < 0.05. Bars represent the mean ± standard error

To investigate the function of *MeGRXC15* in plant, we overexpressed this gene in *Arabidopsis*. Three independent lines of *MeGRXC15-OE* transgenic *Arabidopsis* were used in ABA and drought tolerance analyses and the transgenic *Arabidopsis* lines contained the empty vector (Fig. 5a) were used as controls. We found that ABA did not affect the seed germination of transgenic plants. We infer that overexpression of *MeGRXC15* may cause ABA insensitivity in *Arabidopsis*. Next, 5-day-old seedlings of *MeGRXC15-OE* transgenic *Arabidopsis* were grown on MS medium supplement with 0 μM (mock) or 5 μM ABA, respectively. After 10 days grown on MS medium, no visible phenotypic differences between *MeGRXC15-OE* and control plants were observed (Fig. 7a). On ABA-supplement medium, the growth of control plants was significantly inhibited, while the growth of *MeGRXC15-OE* plants was less inhibited (Fig. 7a). The rosette diameter of *MeGRXC15-OE* plants was ~ 28% higher than that of control plants (Fig. 7b). Also, the primary root of *MeGRXC15*-OE plants was ~ 30% longer than that of control plants (Fig. 7c). Our data support the possibility that overexpression of *MeGRXC15* caused ABA insensitivity in *Arabidopsis*.

To further investigate the roles of *MeGRXC15* in drought tolerance, the control and three independent lines of *MeGRXC15-OE* plants were grown in soil in one pot under normal conditions. After planted in soil for 21 days, the plants were treated by withholding water (Fig. 7d). When exposed to water deficit for 21 days, all treated plants displayed severe wilting (Fig. 7d). The stressed plants were re-watered for five more days and then calculated the survival rate. The *MeGRXC15-OE* lines display a significantly lower survival rate than control plants (Fig. 7d). This indicates that overexpression of *MeGRXC15* caused drought hypersensitivity in *Arabidopsis* under soil culture conditions. We monitored water loss rates of leaves from transgenic *Arabidopsis*. The leaves of *MeGRXC15*-OE plants lost ~ 10% more water than leaves of control plants did at seven hours after excised (Fig. 7e). However, the *MeGRXC15* overexpression shows no effects on biosynthesis of endogenous ABA in *Arabidopsis* under normal and drought conditions (Fig. 7f).

Drought stress also leads to obviously physiological changes in plants. We monitored three stress responsive metabolites including proline, soluble sugar, and malondialdehyde (MDA). We found that overexpression of *MeGRXC15* slightly affected proline and soluble sugar content, but dramatically increased MDA content in *MeGRXC15*-OE *Arabidopsis*, compared to that in the control plants under normal conditions (Fig. 7g, h, i). Prolonged (15 days) drought significantly induced the content of proline, soluble sugar, and MDA in both control and *MeGRXC15*-OE *Arabidopsis* (Fig. 7g, h, i). However, after drought treatment, only MDA content showed significant difference between *MeGRXC15*-OE and control plants. We found ~ 25% more MDA content in *MeGRXC15*-OE plants than that in control plants after drought treatment for 15 days (Fig. 7i). MDA is considered to be the final product of lipid peroxidation in the plant cell membrane and is an important indicator of membrane system injuries and cellular metabolism deterioration [34]. Our results indicate that overexpression of *MeGRXC15* led to cell damage sensitivity to drought in *Arabidopsis*. It may partly explain the drought hypersensitivity in *MeGRXC15*-OE *Arabidopsis*.

### *MeGRXC15* regulates a group of genes involved in stress response and ABA, JA/ET signalling in *Arabidopsis*

**Fig. 8:**
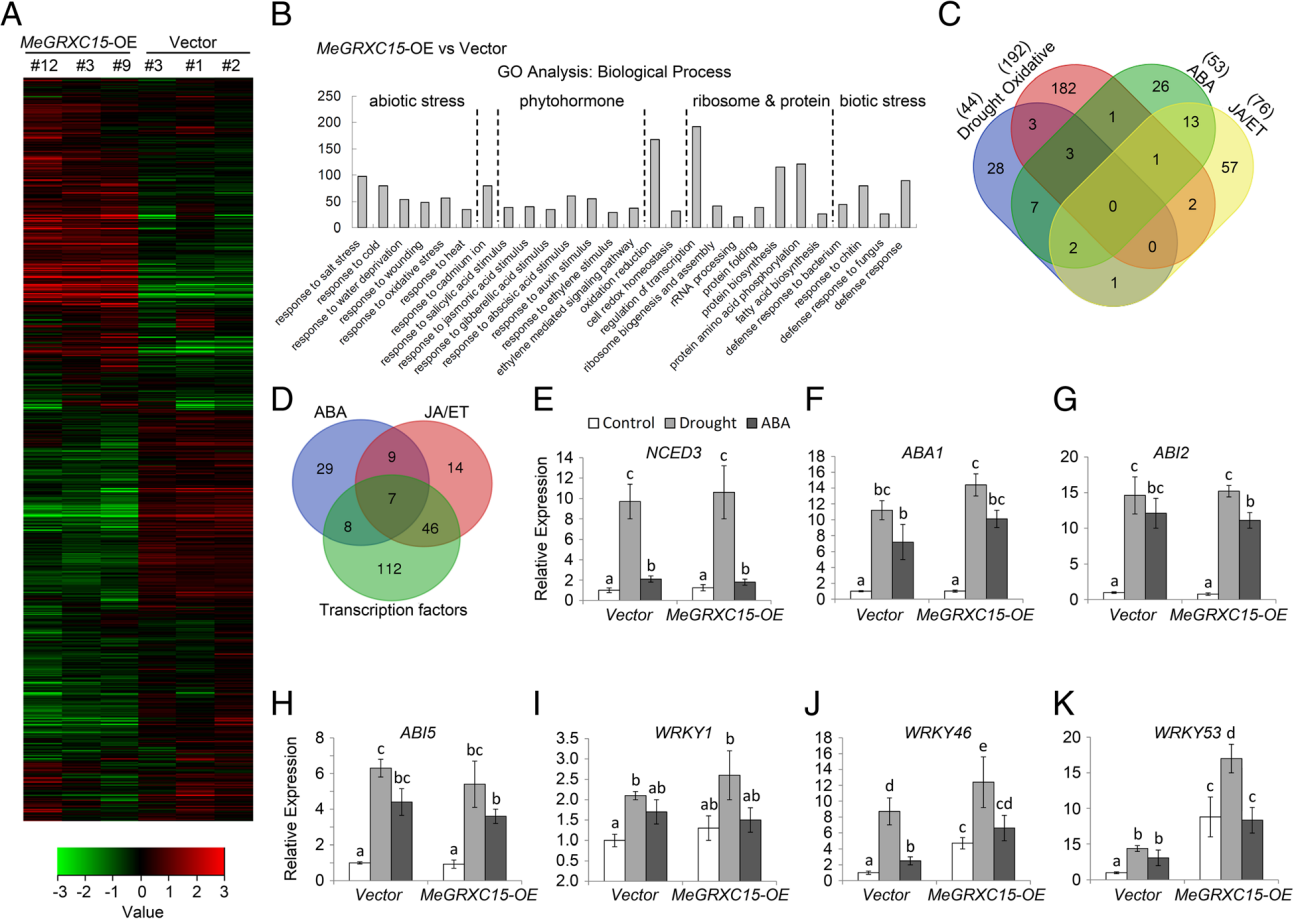
Gene expression profiles in transgenic *Arabidopsis.* **a** Heat map represent gene expression differences between *MeGRXC15-OE* and control (Vector) plants. The data was processed and normalized as described in Materials and Methods. Hierarchical clustering of significantly expressed genes is displayed by average linkage. The figure was drawn by TreeView software. **b** GO analysis of *MeGRXC15-OE* induced genes in *Arabidopsis*. Comparison of GO terms identified from the differentially expression genes identified in SAM analysis. GO tags were selected according to the significance (*p*-value < 10^− 5^). Numbers on y-axis indicate gene numbers of the GO tag. **c** Venn diagram showing the overlap between *MeGRXC15*-OE regulated genes in response to different stress and signals. **d** Venn diagram showing the *MeGRXC15*-OE induced transcription factors which involve in ABA and JA/ET signalling. Expression analysis of *NCED* (**e**), *ABI1* (**f**), *ABI2* (**g**), *ABI5* (**h**), *WRKY1* (**i**), *WRKY46* (**j**), and *WRKY53* (**k**) in transgenic Arabidopsis under drought and ABA treatments. Expression levels of target genes were normalized against vector control. Biological triplicates were averaged and significance of difference between treatments and control was analyzed using the Duncan’s multiple range tests. Different letters represent a significant difference at *p* < 0.05. Bars represent the mean ± standard error

To understand the effects of the *MeGRXC15* overexpression on gene expression in *Arabidopsis*, a microarray analysis was performed using the Affymetrix Arabidopsis ATH1 Genome Array. Three independent lines of *MeGRXC15-OE* and control *Arabidopsis* grown in soil under normal conditions were used. We found that transcription levels of 2674 genes were altered significantly (with more than a twofold change; *P* value < 0.05) in *MeGRXC15-OE* lines compared with control lines under normal conditions (Additional file 2: Table S2). 1264 genes were up-regulated, whereas 1410 genes were down-regulated. The relative expression levels of these genes were shown by the heat map (Fig. 8a). Gene ontology (GO) analysis shows that many stress-responsive genes are affected by *MeGRXC15-*OE *Arabidopsis* (Fig. 8b). 27 more abundant GO categories (q-value < 10^− 5^) including categories of response to abiotic, biotic stress, and phytohormone stimulus in *MeGRXC15*-OE *Arabidopsis* are exhibited here. Interestingly, nearly two hundred transcription factors were affected by *MeGRXC15-*OE *Arabidopsis* (Fig. 8b). We found that 192 oxidative stress-related, 44 drought-related, and 53 ABA-related genes were significantly altered in *MeGRXC15-OE* plants. Nevertheless, there are three members overlapping with the genes involved in response to drought, oxidative stress and ABA (Fig. 8c), indicating that a specific regulatory mechanism dependent on ABA-oxidative crosstalk conferred by MeGRXC15 is presented in response to drought. Moreover, three drought-related and three oxidative stress-related genes overlapping with genes that involved in JA/ET signal transduction respectively (Fig. 8c), suggesting the *MeGRXC15* may play roles in drought response depending on regulation of JA/ET pathway. There are seven transcription factors overlapping with the genes involved in response to ABA or JA/ET (Fig. 8d). This suggests the regulatory roles of *MeGRXC15* in ABA and JA/ET crosstalk.

To clarify gene expression in *MeGRXC15*-OE *Arabidopsis* during drought and ABA treatments, we analyzed genes that are related to ABA- or drought-responses, including *NCED3, ABI1, ABI2, ABI5, WRKY1, WRKY46,* and *WRKY53.* The qPCR results show that expression of *NCED3, ABI1, ABI2*, and *ABI5* was not affected by *MeGRXC15*-OE *Arabidopsis* under normal conditions (Fig. 8e-h). When exposed to drought or ABA, expression of these four genes was up-regulated in both control and *MeGRXC15*-OE plants (Fig. 8e-h). We found that expression of *WRKY1, WRKY46*, and *WRKY53* was up-regulated by *MeGRXC15*-OE *Arabidopsis* under normal conditions (Fig. 8i-k). Furthermore, drought or ABA treatments both up-regulated *WRKY1, WRKY46,* and *WRKY53* transcription in control plants (Fig. 8i-k). Likewise, drought significantly up-regulated these three *WRKY*s transcription in *MeGRXC15*-OE plants (Fig. 8i-k). However, ABA treatment did not affect transcription of *WRKY1* and *WRKY53* (Fig. 8i-k), it only slightly up-regulated *WRKY46* transcription in *MeGRXC15*-OE plants (Fig. 8 i-k).

### MeGRXC15 interacts with TGA5 or MeTGA074

**Fig. 9:**
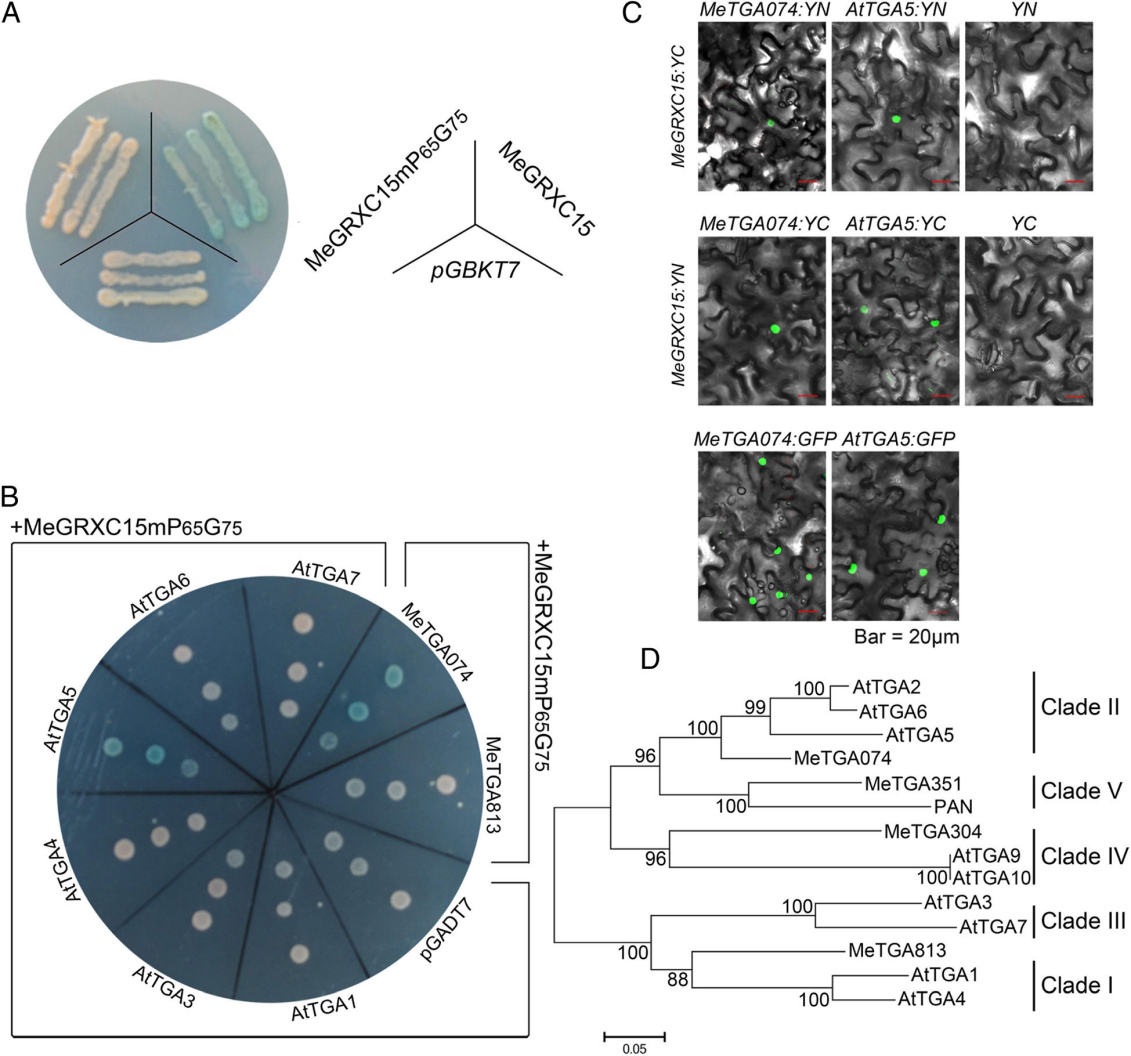
Identification of protein interacts with MeGRXC15 in cassava and *Arabidopsis*. **a** Autonomous transactivation analysis of MeGRXC15 in yeast. MeGRXC15mP_65_G_65_ indicate mutant in MeGRXC15 GSH binding site. **b** Analysis of interaction between MeGRXC15 mP_65_G_65_ and TGA factors by yeast two-hybrid system. **c** BiFC analysis of the interactions between MeGRXC15 and TGAs identified by yeast two-hybrid system in transiently transformed *N. benthamiana* leaves. Green fluorescence in nucleus was detected for interactions of MeGRXC15 with MeTGA074 and AtTGA2, respectively. As a negative control, co-expression of MeGRXC15:YN with free YC, and MeGRXC15:YC with free YN failed to reconstitute a fluorescent YFP chromophore. Expression of MeTGA074:GFP and AtTGA2:GFP in transiently transformed *N. benthamiana* as positive controls. **d** Phylogenic analysis of TGA factors from *Arabidopsis* and cassava. A neighbor-joining tree was constructed with MEGA5.0 software based on sequences alignment with ClustalX

Several CC-type GRXs play roles in organ development or plant defense via interaction with TGA transcription factors [15, 17, 35, 36]. TGA transcription factors regulate genes that involved in both biotic and abiotic stress [37]. It is necessary to identify the interactors of MeGRXC15 in *Arabidopsis* and cassava. We fused MeGRXC15 with the GAL4 DNA-binding domain (BD) in *pGBKT7* (Clontech) and then transformed the resulting construct into yeast strain Y187. The *pGBKT7* vector was used as negative control. However, yeast cells harboring *MeGRXC15:pGBKT7* activated X-α-gal on SD/−Trp / X-α-gal medium (Fig. 9a), suggesting that MeGRXC15 has transcriptional activation ability. CC-type GRXs need to interact with glutathione (GSH) to catalyze essential biosynthesis reactions by its redox regulation [25]. Therefore we created a MeGRXC15 mutant by replacing the GSH binding site. As is shown in Fig. 9a, the MeGRXC15 mutant MeGRXC15mP_65_G_75_ did not activate X-α-gal on the medium. This suggests that the GSH binding site is required for the transcriptional activation ability of MeGRXC15. A possible explanation is that MeGRXC15 may bind and modify the transcription factor depending on GSH in yeast.

Subsequently, six TGA transcription factors including TGA1, 3, 4, 5, 6, 7 in *Arabidopsis* and two TGA transcription factors (MeTGA074 and MeTGA813) in cassava were respectively fused with GAL4 activation domain (AD) sequence in pGADT7 (Clontech). The resulting AD:TGA constructs and BD:MeGRXC15mP_65_G_75_ were pairwise co-transformed into yeast Y187, respectively. Yeast cells that harbored both *AD:TGA* and *BD:MeGRXC15mP*_*65*_*G*_*75*_ pair plasmids were grown on SD/ -Trp/ -Leu/ X-α-gal medium. The yeast cells containing pairwise plasmids AD:TGA5 / BD:MeGRXC15mP_65_G_75_ and AD:MeTGA074 / BD:MeGRXC15mP_65_G_75_ activated X-α-gal (Fig. 9b). This suggests that MeGRXC15 could respectively interact with TGA5 or MeTGA074.

To further investigate the interactions between MeGRXC15 and AtTGA5 / MeTGA074 *in planta*, we employed Bimolecular Fluorescence Complementation (BiFC) analysis. Nuclear fluorescence co-expression of *MeGRXC15* and *AtTGA5/MeTGA074* was detected in epidermal cells (Fig. 9c). The *in planta* nuclear interactions of MeGRXC15 with AtTGA5 / MeTGA074 suggest that this CC-type GRX might function in *Arabidopsis* and cassava by nuclear interaction with AtTGA5 / MeTGA074. We created a phylogenetic tree based on TGA protein sequences in *Arabidopsis* and cassava (Fig. 9d). We found that MeTGA074 is a member of clade II TGAs, closely related to AtTGA5. Together, our data suggest that *MeGRXC15* may regulate drought response via interaction with AtTGA5 / MeTGA074.

## Discussion

In *Arabidopsis*, only four of 21 CC-type GRXs (*GRXC7/ROXY1, GRXC8/ROXY2, GRXC9/ GRX480/ROXY19*, and *GRXS13*) were functionally characterized by genetic approaches [14–16, 25]. With such short knowledge identifying of the genomic and EST sequences in several plant species is a promising approach, which may allow expanding the knowledge of plant GRXs by the comparative and evolutionary analysis. Herein, we identified 38 putative GRX genes from cassava genome (Fig. 1), they are classified in five subgroups as in *Arabidopsis* and rice [6, 7]. CC-type GRXs are a land plant specific subgroup of the GRX family, derived from the CPYC subgroup and expanded from basal to higher land plant mainly through paleopolyploidy duplication and tandem duplication events [11]. Our result demonstrated that all cassava CC-type GRXs evolved from three cassava CPYC GRXs (Fig. 1). And we found *MeGRXC1* and *MeGRXC2*, *MeGRXC3* and *MeGRXC4* are two pairs neighboring genes in cassava chromosome 1 (Table 1). Also *MeGRXC8* and *MeGRXC9* are neighboring genes in chromosome 5, *MeGRXC14* and *MeGRXC15* are neighboring genes in chromosome 15 (Table 1). These results indicate that tandem duplication events contributed to the expansion of CC-type GRXs in cassava.

To date, no CC-type GRX was characterized as a regulator of drought response in ABA dependent manner. Based on the RNA-seq and qPCR data, we found that six CC-type GRX genes were induced by drought stress in leaves of both Arg7 and SC124 (Fig. 3). Under drought stress, ABA concentrations increase and, in turn, induce gene expression [38]. Overexpression of the rice CC-type GRX *OsGRX8* enhances tolerance to ABA and abiotic stresses in *Arabidopsis,* but the expression of this gene was induced by auxin, instead of ABA in rice [18]. However, exogenous ABA application induced the expression of the six drought-responsive CC-type GRX genes in leaves of both Arg7 and SC124 (Fig. 4), suggesting that CC-type GRXs regulated drought response probably in an ABA-dependent manner in cassava. This is further supported by our data showing that overexpression of *MeGRXC15* in *Arabidopsis* resulted seedling development insensitivity to ABA (Fig. 7a) and induced overexpression of several genes which involved in the ABA signalling (Fig. 8).

In *Arabidopsis*, seven *ROXY* members under the control of *ROXY1* promoter could complement the* roxy1* mutant [35]. This indicates that the expression pattern is particularly important for the function of *ROXY* genes. When exposure to prolonged drought stress, the Arg7 plant display faster senescence in older leaves than the SC124 plants [2]. *MeGRXC3, C7*, and *C15* shown fairly consistent difference in expression levels between Arg7 and SC124 in both drought and ABA treatments (Fig. 3, Figs 4 and 6), suggesting that the expression patterns of these genes are correlated with the different response of leaves in Arg7 and SC124 under drought stress. The *GRXS13* is a CC-type GRX, which could be induced by oxidative stress in *Arabidopsis*, repression of this gene resulted higher accumulation of the superoxide anion O^2−^ [16]. High ROS accumulation is required for abscission zone formation in cassava during drought stress [3]. Our data also shown that *MeGRXC15* has highest expression levels in abscission zone (AZ) at D8 in both two cassava cultivars (Fig. 6), indicating the expression of *MeGRXC15* maybe correlated to ROS accumulation. ABA induced ROS production is a required process in plant drought response [39, 40]. The gene expression profile in transgenic *Arabidopsis* shows that *MeGRXC15* overexpression induced three genes overlapping with the genes involved in response to drought, oxidative stress and ABA (Fig. 8c), suggesting that *MeGRXC15* regulates drought response likely through ABA and ROS signalling pathway. Overexpression of *MeGRXC15* did not affect endogenous ABA synthesis (Fig. 7e) and *NCED3* transcription (Fig. 8e), while it affected *WRKY46* and *WRKY53* transcription in *Arabidopsis* (Fig. 8j, k). In *Arabidopsis*, *WRKY1, WRKY46*, and *WRKY53* negatively regulate drought tolerance by inhibition of ABA-induced stomatal closure [41–43]. Additionally, drought dramatically up-regulated *WRKY46* and *WRKY53* in *MeGRXC15*-OE plants (Fig. 8j, k), this may partly ascribe to the drought sensitivity of these plants. *WRKY46* and *WRKY53* are also involve in signalling transduction of other phytohormone, such as Brassinosteroid [44], Jasmonic acid and Salicylic acid [45]. ABA treatments did not affect the expression of *WRKY46* and *WRKY53* in transgenic *Arabidopsis* (Fig. 8j, k), indicating that MeGRXC15 may regulate *WRKY*s through an ABA-independent pathway during drought response. Furthermore, regulation on *WRKY*s expression under ABA treatment perhaps contributes to the ABA insensitivity in *MeGRXC15*-OE *Arabidopsis*.

The nuclear localization of ROXY1 is required for its function in petal development [35]. However, not all members of CC-type GRX subgroup have the same subcellular localization, unlike ROXY1, ROXY18 and ROXY20 are localized in cytosol [7]. All of our six drought-responsive CC-type GRXs are located in both nucleus and cytosol (Fig. 5b), suggesting the possibility that these genes could function in nucleus. The nuclear functions of ROXYs in *Arabidopsis* are partly dependent on its interaction with TGA transcription factors [15, 35, 36]. In *Arabidopsis*, TGA transcription factors have been classified to five subgroups, clade I, II, III, IV, and V. TGA2, 5, 6 are members of clade II TGAs, which are essential activators of jasmonic acid/ethylene-induce defense responses [15, 46–48] and act as key regulators in plant responses of abiotic stresses such as drought, cold, and oxidative stress [37]. *Arabidopsis* CC-type GRX GRX480/ROXY19 could interact with TGA2, 5, 6 [15]. TGA2 could interact with GRXS13, and act as repressors of *GRXS13* expression in response to biotic stress [14]. Here, we found that MeGRXC15 could interact with *Arabidopsis* TGA5 and cassava MeTGA074 in the nucleus respectively (Fig. 9b, c). In *Arabidopsis*, GRX480 regulated the expression of *ERF* (*Ethylene Response Factor*) factors through interaction with TGA2/5/6 [15, 49]. We found 53 transcription factors including ERFs were induced by *MeGRXC15* in *Arabidopsis* (Fig. 8d). A NES (Nuclear Export Signal) could be tagged to MeGRXC15 to eliminate its nuclear localization to investigate whether the MeGRXC15 regulates the transcription factors through nuclear interaction with TGA5 in *Arabidopsis*. It will be of interest to further study the mechanism by which MeGRXC15 respond to drought in ABA dependent manner via interaction with MeTGA074 in cassava.

In our study, when MeGRXC15 was fused to GAL4 binding domain (BD), the fusion protein exhibited strong autonomous transactivation activity in yeast (Fig. 9a), indicating that MeGRXC15 could recruit transcription factor in yeast nucleus and probably generate a complex protein like GAL4BD-MeGRXC15-TF (Activation Domain). Thus, the recombination protein was able to function as a transcription factor promoting the transcription of reporter gene in yeast strain Y187. Substitution mutants in GSH binding site of MeGRXC15 caused autonomous transactivation activity loss in yeast (Fig. 9a). The GRXs are generally reduced by GSH, and the GHS binding ability is required for the function of ROXY1 and ROXY2 in *Arabidopsis* [25]. The CC-type GRXs interact with TGA transcription factors dependent on its C-terminal L**LL and ALWL motifs [15, 36]. The mutation in GSH binding site probably will not affect the CC-type GRX interaction with TGA transcription factors. However, the ROXY1 negatively regulates the PAN activity, and positively regulates the other TGAs activity during the petal development in *Arabidopsis* [35]. Thus, modification of the GSH binding site of MeGRXC15 may affect its regulation on TGAs activity and therefore caused transcriptional activation losing of GAL4BD-MeGRXC15-TF complex protein.

## Conclusion

Our study demonstrates that CC-type GRXs may function in ABA-mediated drought signalling in cassava. As a CC-type GRX, MeGRXC15 could interact with *Arabidopsis* TGA5 or cassava MeTGA074. Overexpression of *MeGRXC15* results drought hypersensitivity in *Arabidopsis*. It will contribute to an enhanced understanding of the specific mechanisms that elucidate the roles of CC-type GRXs involved in drought response in cassava.

## Methods

### Bioinformatics analysis

The protein sequences of cassava GRXs were predicted using a TBLASTN search against the cassava genome database in Phytozome (https://phytozome.jgi.doe.gov/pz/portal.html, *Manihot esculenta v6.1*) with the protein sequence from *Arabidopsis* GRXs as a query. All *Arabidopsis* GRX protein sequences were downloaded from GenBank. Multiple sequence alignments were conducted using ClustalW [50] based on GRX protein sequences in Additional file 1: Table S1. An unrooted phylogenetic tree showing cassava GRXs and *Arabidopsis* GRX family was generated via the neighbor joining method using MEGA5.0 [51]. Editing of aligned sequences of cassava CC-type GRXs was performed using AlignX (Vector NTI suite 10.3, Invitrogen).

### Transcriptome data analysis

For drought-responsive CC-type GRXs identification, we used our previously reported RNA-seq data [24]. We used data that included two tissues (leaf and root) under drought treatment and a control. The accession number of RNA-seq data is listed in Additional file 2: Table S2. Gene expression levels were normalized using FPKM. We selected the data of CC-type GRX genes (Additional file 3: Table S3), and generated a heat map and hierarchical clustering using Cluster 3.0.

### Drought and ABA treatments on cassava

Two cassava cultivars, Arg7 and SC124, were used in this study. Stems of cassava Arg7 and SC124 were cultured in same pots (36 cm in diameter × 30 cm in height) containing well-mixed soil (nutrient soil: vermiculite: sand, 1:1:1) for 90 days in greenhouse at the Institute of Tropical Bioscience and Biotechnology (Haikou, China). For drought treatment, plants were treated by withholding water for eight or 14 days. Different tissues were collected from three Arg7 and SC124 plants at eight or 14 days after withholding water and 24 h after re-watering at the ending of treatment. Plants watered as normal were used as controls. Different tissues including Functional Leaf (FL), New Leaf (NL), Petiole (P), Stem (S), Abscission Zone (AZ) and Root ® from each plant were collected. For ABA treatments, mature leaves with petiole were excised from Arg7 and SC124 plants, treated by dipping the leaves in water (control) or in water with 20 μM ABA. The samples were collected after treated for 0, 0.5, 1, or 2 h.

### Quantitative real-time PCR (qPCR) analysis

Total RNA was isolated from cassava or Arabidopsis leaves using RNAprep Pure Plant Kit (TIANGEN). The cDNA synthesis was performed with FastQuant RT Kit (TIANGEN). Expression analysis of CC-type GRXs in cassava after drought and exogenous ABA treatment was performed by qPCR with gene-specific primers (Additional file 2: Table. S4). All qPCR reactions were carried out in triplicates, with SYBR® Premix Ex Taq™ II Kit (Takara) on StepOne™ Real-Time PCR system (Applied Biosystems), and the comparative ΔΔCT method employed to evaluate amplified product quantities in the samples.

### Protein subcellular localization

Full-length coding sequence without stop-codon of *MeGRXC3, C4, C7, C14, C15,* and *C18* was isolated from cDNA of drought stressed leaves by RT-PCR respectively. Fragments were identified by sequencing and fused to *GFP* in front of the *CaMV 35S* promoter in the modified plant expression vector *pG1300* (*eGFP:pCAMBIA1300*) to make *35S:MeGRXC3:GFP*, *35S:MeGRXC4:GFP, 35S:MeGRXC7:GFP*, *35S:MeGRXC14:GFP, 35S:MeGRXC15:GFP*, and *35S:MeGRXC18:GFP*. The 5’UTR of ATADH gene was inserted between 35S promoter and MeGRX coding sequence to enhance the expression of *MeGRX:GFP*. The resulting constructs and empty vector were transformed into *Agrobacterium LBA4404*. Leaves from four-week-old *Nicotiana benthamiana* plants were transformed by infiltration of *Agrobacterium* cells (OD_600_ = 1.2) harboring appropriate DNA construct using 5-mL syringe without needle. The empty vector *(GFP)* and *35S:MeHistone3:GFP (H3:GFP)* were used as the positive controls. After three days, infiltrated *N. benthamiana* leaves were imaged for reconstitution of GFP fluorescence by confocal laser scanning microscope (Olympus FluoView FV1100).

### Generation of *MeGRXC15-OE* transgenic *Arabidopsis*

Wild type (Col-0) *Arabidopsis* plants for transformation were grown in 12 h light/12 h dark at 20–23 °C until the primary inflorescence was 5–15 cm tall and secondary inflorescence appeared at the rosette. *Arabidopsis* was transformed using the floral dip method [52] and *A. tumefaciens* strain *LBA4404* carrying the DNA constructs *35S:MeGRXC15:GFP* and the *pG1300* empty vector control, respectively. More than three homozygous lines of each construct were selected for further phenotypic analyses. The *MeGRXC15:GFP* fusion protein subcellular localization in transgenic *Arabidopsis* epidermal cell was examined for reconstitution of green fluorescence by confocal laser scanning microscope (Olympus FluoView FV1100).

### ABA tolerance assays of transgenic *Arabidopsis*

To study the response of *MeGRXC15-OE* transgenic plants to ABA, 5-d-old seedlings were transferred to MS medium containing with 0 μM (mock) and 5 μM ABA grown for 10 days. Rosette diameter, primary root length and lateral root number were measured. The transgenic plant that contained *pG1300* vector was used as the empty vector control.

### Drought stress tolerance assays of transgenic *Arabidopsis*

Post-germinated seedlings of *MeGRXC15-OE* and empty vector transgenic plants were grown in soil in one pot for 15 days under normal conditions. For drought stress, the plants were treated by water withholding for 21 days, then re-watering. Survival rates were calculated at five days after re-watering. Proline and soluble sugar, indicator of the drought response in plants, were measured. Lipid peroxidation in transgenic *Arabidopsis* leaf tissues was measured in terms of malondialdehyde (MDA) in the samples as described in reference [5] during drought stress. For water loss rate measurement, excised leaves from 28-d-old unstressed transgenic plants were kept on plastic dishes at room temperature. Their weight was measured after one hour, up to seven hour followed by calculation of water loss percentage.

### Determination of endogenous ABA content

Endogenous ABA content was determined by extraction and detection using LC-ESI-MS/MS according to methods described previously [53]. 28-d-old leaves from five control or drought stressed plants of each line were mixed to constitute one biological replicate. 0.1 g mixed leaf sample was extracted with 1.5 mL methanol formic acid solution (Methanol: formic acid: water = 7.8: 0.2: 2). Results from three biological replicates were averaged.

### Microarray analysis of transgenic *Arabidopsis*

Microarray experiments were conducted using Affymetrix Arabidopsis ATH1 Genome Array. Experiments were performed as three biological repeats using cDNAs prepared independently from three individual homozygous lines of *MeGRXC15* overexpression *Arabidopsis* that were phenotypic analyzed in plant growth. The transgenic *Arabidopsis* plants that carried the *pG1300* empty vector were used as controls. The experiments and data analysis were performed under the instruction of Affymetrix. Total microarray data were deposited in the NCBI GEO database with the accession number: GSE81136 (MeGRX232-OE). Gene ontology (GO) analyses for significant enrichments of various categories (Additional file 2: Table S5) were performed using MAS 3.0 (http://bioinfo.capitalbio.com/mas3/). The Venn diagrams were created by online tool (http://bioinformatics.psb.ugent.be/webtools/Venn/).

### Identification and phylogenetic analysis of TGA transcription factors

The *Arabidopsis* TGA transcription factors protein sequences were download from GenBank database. The cassava TGA transcription factors were identified using TBLASTN against the Phytozome database website (https://phytozome.jgi.doe.gov, *Manihot esculenta v6.1*) with the protein sequences from *Arabidopsis* TGA transcription factors. Four TGA transcription factors were cloned with the accession number Manes.04G157200.1 (MeTGA074), Manes.04G004100.1 (MeTGA304), Manes.14G099100.1 (MeTGA351), and Manes.12G140100.1 (MeTGA813). An unrooted phylogenetic tree showing cassava and *Arabidopsis* TGA transcription factors was generated based on protein sequences (Additional file 3: Table S6) with a neighbor joining method using MEGA5.0 [51].

### Transactivation analysis and yeast two hybrid assay

Before analyzing the interaction between MeGRXC15 and TGA transcription factors, an autonomous transactivation analysis was performed in yeast strain Y187. The MeGRXC15 was in frame fused to GAL4 BD (binding domain) in *pGBKT7*, and then transformed into yeast Y187. Because MeGRXC15 shows “autonomous transactivation” in yeast, a MeGRXC15 GSH binding site mutant *MeGRXC15mP*_*65*_*G*_*75*_ was produced by replacing P_65_AVFIGGILVG_75_ to A_65_AVFIGGILVA_75_. Next, for identification the interaction between MeGRXC15 and TGA transcription factors, a yeast two-hybrid assay has been performed in yeast strain Y187 based on the Matchmaker ™ GAL4 two-hybrid system 3 manual (Clontech). The *MeGRXC15* GSH binding site mutant DNA construct *MeGRXC15mP*_*65*_*G*_*75*_*:pGBKT7* was used as bait. The cDNA sequences of TGA transcription factors from *Arabidopsis* and cassava were introduced into the pGADT7, respectively in frame fused to GAL4 activation domain (AD). The *MeGRXC15mP*_*65*_*G*_*75*_*:pGBKT7* and *TGA:pGADT7* constructs were pairwise co-transformed into yeast strain Y187. The presence of transgenes was confirmed by growth on SD/ -Trp/−Leu plates. Interactions between two proteins were checked by examining *β*-galactosidase activity as the manual instructed.

### Bimolecular fluorescence complementation analysis

To confirm the interactions between MeGRXC15 and TGA2 / MeTGA074 factors, a bimolecular fluorescence complementation assay was performed using the *N.benthamiana* transient system as previously report [54]. The full-length coding sequence without stop-codon of *MeGRXC15* was in frame fused to N- or C-terminus to yellow fluorescent protein (YFP) fragments (YN/YC) respectively to produce *35S:MeGRXC15:YN:pBiFC* and *35S:MeGRXC15:YC:pBiFC.* The full-length coding sequence without stop-codon of *TGA2* and *MeTGA074* were in frame fused to YC or YN respectively to produce *35S:TGA2:YC:pBiFC, 35S:TGA2:YN:pBiFC, 35S:MeTGA074:YC:pBiFC,* and *35S:MeTGA074:YN:pBiFC.* The resulting constructs were then introduced into *A. tumefaciens LBA4404* strains. Constructs were pair-wise transiently expressed in epidermal cells of tobacco leaves. Three days after agrobacterium co-transformation of leaves, reconstitution of YFP fluorescence was examined by confocal microscopy using GFP filter. Then the assays were performed as the method of proteins subcellular localization described. As positive controls, full-length green fluorescent protein (eGFP) was tagged to the C-terminus of TGA2 and MeTGA074 respectively, transiently expressed in tobacco leaves.

## Additional files

Additional file 1: **Table S1.** The protein sequences of GRX from cassava and Arabidopsis. (DOC 61 kb)

Additional file 2: **Table S2.** The accession number of cassava drought related transcriptome data. **Table S3.** The RNA-seq data of GRXs in drought stressed cassava. **Table S4.** The list of primers. **Table S5.** GO results of MeGRXC15 regulated genes in transgenic *Arabidopsis*. (XLS 287 kb)

Additional file 3: **Table S6.** Protein sequences of TGA transcription factors from cassava. and *Arabidopsis. (DOC 35 kb)*

## Abbreviations

ABA: Abscisic acid
AD: Activation domain
Arg7: Argentina 7
BD: Binding domain
BLAST: Basic local alignment search tool
ET: Ethylene
GFP: Green fluorescent protein
JA: Jasmonate acid
NES: Nuclear export signal
qPCR: Quantitative real-time polymerase chain reaction
ROS: Reactive oxygen species
SC124: South china 124
YFP: Yellow fluorescent protein
