# Supplementary material for: Identification and characterization of drought-responsive CC-type glutaredoxins from cassava cultivars reveals their involvement in ABA signalling
Source: BMC Plant Biol. 2018 Dec 4;18:329. doi: 10.1186/s12870-018-1528-6 (PMC6280520; doi:10.1186/s12870-018-1528-6)
Supplement: Supplementary file 1 — Table S1. The protein sequences of GRX from cassava and Arabidopsis. (DOC 61 kb) [file 12870_2018_1528_MOESM1_ESM.doc]

Identification and characterization of drought-responsive CC-type glutaredoxins from cassava cultivars reveals their involvement in ABA signalling.

Meng-Bin Ruan^1,2,†,*^, Yi-Ling Yang^3,†^, Xin Guo^4^, Bin Wang^4^, Xiao-Ling Yu^1,2^, Kai-Mian Li ^5^, and Ming Peng^1,2,*^

Table S1. Protein sequences of glutaredoxin in cassava and *Arabidopsis*.

>cassava4.1_028088m

MGCISSKLCRKQLQQDIIVNNGGRECFNHVVSLTSSTYGALKLDINEKLQQQQDAAATEEEPIKEPVSESKKMQQSSPPREDPEVINAWELMKDLEDGGIPISNHPKKSPKSRALLRGFADMDARTPLKFLNQIGSPRKARTFGGKENKVKRVSEFISPRPVLKANNSSGKSSKAVLRLSYPVKASPVSAKTENAGCDDSEDSSRRRRSFSPLFDPELVALYEKELSEEEGQIKRIISLTPRPQKSKNARELESFLHSFEQKCPPGGDNAVVIYTTTLRGIRKTFEDCNTVRSIIDSYHIHIIERDISMDSGFKEEIRRLMGSMEVKVPLVFVRGRLIGGADQVVKLEEEGKLGILFDGIPKRLSGGCEVCAGFRFVMCKECNGSCKILNQEQKKMVKCGECNENGLIHCPICC*

>cassava4.1_032936m

MQYQYKYQHQQQGAESSSWGYYVPAARGMVGSDPLERVVRLASESAVVIFSISSCCMCHAVKRLFCGMGVNPTVYELDQDPRGKEIERALMRLLGNYSSAVPVVFIGGKLIGAMDRVMASHINGTLVPLLKEAGALWL*

>cassava4.1_024232m

MDRVTQLASERAVVIFSRSTCCMCHAIKTLLCDFGVNPTVHELDEIPRGREIEQALSRLGSPTLPAVFIGGILVGGANEVMSLHLNRSLIPMLRRAGALWV*

>cassava4.1_026496m

MDRVAKLASQKAVVIFSKSSCCMCHAIKRLFYEQGVSPAIYELDEDPRGKEMEWALMRLGCNPSVPAVFIGGKFVGSANTIMTLHLNGSLKKLLREAGAIWL*

>cassava4.1_028275m

MGCASSKQKRCRHCHAPYSPIPRSYSMHVVHPPEKKGDSYHVVALTSTTLGSLALDSPANYKDNVSAAIDILAAIENDRENSSVCDGQSKDLKNKEFSVGLIEAKSWSNKIQAKIPKIVPRTPVRTPPGEPETINTWELMAGLEEDDGSVHLPHRFRSFSFDVSRDPSPVLDSPRISGTKFSPNKPQTDPKPLWLQLADEEANSKFPQLDSQIFCSPRKSLQDLSPTHPFYLKPSDGEKQQSPPPSHPTAGNDPIKEFSKSGKEKVVVYFTSLRGVRKTYEDCCHVRVILKTLGVRIDERDVSMHSGFKEELRELLGEGYSGGLPKVFIGRKFIGGAEEIRRMHEEGELEKAVEECEMVEDDGGNCNGACEACGDIRFVPCETCSGSCKIYYEREEEEEEEEEDDGDEGEYGFQRCPDCNENGLIRCPICCY*

>cassava4.1_017658m

MARPLSNILLKGIAGLPAVRVRSTSTVFRSFNCNGMRYSTTVPNDPDTHEDFQPNNKLESSGMSLKDIVEQDVKDNPVMIYMKGVPDFPQCGFSSLAVRVLKHYNIPLSARNILEDPELKSAVKSFSNWPTFPQIFIKGEFIGGSDIILNMHQSGELKEKLQDVATSQKSE*

>cassava4.1_031237m

MWLEWLRSPSRAHTSPRQPPEPPSPRYFSSSSFKDINAILLEEENRSKSQPQTPRRPSIFHRASPLYRHHRNRSKTFIISPPPNQDDHKIILYFTSLGIVRRTLEDCRTVRSILRGFHVPIDERDLSMDAEYLDEIQMISASKKVRLPAVFLGGKYLGGAEEINEMNESGELSKLIGGLPFVENNIKIKFNSVCDVCGGLRYVLCAQCNGSHKIYSEKYGFRTCTSCSVNGLIKCGLCYPVQCKRSD*

>cassava4.1_008955m

MGCSASKSNTLDPKSHHLQENFSPESSAFTSFSSPVPRALSLQTPLVHHLPSKKGDSHHLVSLTSTTYGSLLLVEPLKSKLNGRNPPDQPLSPPKFTKNTHKTHVPADPGKSFSPDSVINTWELMDGLDDDLDFEMGDSLKPKSSFSDHAVQVSLKSSSSQRLGFDESIKKLQDSFDSEKSESFSSSKPLWKHLSEESFLSKMDPNVVSSYRRALSSRQLGYTKETTIVARSVGSSPMNPLPTNNNGFLSHNTEDKIVLYFTSLRGIRKTYEDCCAVRMIFRGFRVPVDEKDISMDASYRKELQSLIKCKAISLPQVFIRGKHIGGVEEIRQLNEAGELANILEGFPVRDPRVVCERCGDARFVPCPNCNGSRKVFDEEEEQLKRCMYCNENGLIRCPGCCCS*

>cassava4.1_018360m

MQEAIPFRTRIPSTAIAGGNRQLSPTGSASSNGRNVLVSANRENYVQKLVLENSVIVFGKSGCCMCHVMKRLLLGLGVNPTVFEVDENEEAAVINELSNISCSKDVGEGEVQFPVVFVGGKLFGGLERVMATHICGELVPILKDAGALWL*

>cassava4.1_028408m

MASSVAINSEPPTSKAMNQFDNPYEIVRHLASSNAVVLFSMSGCCMCTVAKRLLFGLGVGPTIIELDHHSAGADIQAVLFQLASDGQQPVPAIFVGGKFLGGIETLMACHINGTLVPLLKDAGALWL*

>cassava4.1_027873m

MEKVTQLASERPVVIFSRSTCAMCHAIKSLLREFGVNPAVYEVDEIPRGREIEQALSRLVSPTLPAVFIGGELVGGANEVMTCHLNRSLIPMLRRAGALWV*

>cassava4.1_024091m

MQGLRRCSNDAVSLDLSPPPPTATSSSSSLSFDAVESVETRIQRLISEHPVIIFSRSSCCMCHVMKKLLATIGVHPTVIELDDHEISALPPPPPSHDNDDAAPRNLAPAVFIGGTCVGGLESLVALHLSGHLVPKLVEVGALWV*

>cassava4.1_034311m

MGCSSSKRVEATLDVYRPPPSSFAVFDINSIQEPWLSLDNSARENQEKSTHLPAPILEKLNKLESDAPHSWEEVSKALEELNNKNSVVAPVRPSTLNPPAKVTAASANPTPEKTPPPRKSSSFHTLEELDAKLSSKPQKELRKTESLRTELKKPEATKIESRVMVEPATESGNVIKPVKENIFILRDRQEREKEGKMANYDKIKWDPLSDFPEKCPPGGGDSVVIYTTSLRGVRRTYEDCNRLRSLLEVHRVVFDERDVSLHGEFLNEIRELLGEEGSVPRVFVKGRYIGGVEKVVELNESGRLGRILNWARVERGVGRQACEGCGGARFMPCLDCGGSCKVIVDGVKERCGKCNENGLVLCPACL*

>cassava4.1_018918m

MQEGLRRCSDQALRLDLTSSSLSFDAVESAETRIQRLISEHPVIIFSRSSCCMCHVMKKLLSTIGVHPTVIELDDHEISALPSPPPSDDVPSPRNLAPAVFIGGTCVGGLESLVALHLSGHLVPKLVEVGALWA*

>cassava4.1_019241m

MGSLLSSTKTTKEETDMALNKAKQISTSAPVVVFSKTYCGYCKRVKQLLTQLGASFRVIELDEESDGAEIQSALAQWTGLRTVPNVFIGGQHIGGCDSTLDKYQKGELLPLLNDAGAIANNSAQL*

>cassava4.1_014058m

MAEFESNMEFKGKSNPSTMATSFFNRSLTIHSSKSSPKDFAPSSSRFYYSFESMKGKVKQLCNIFESSREPKSVLHETQSQSLTQASKLKSVKSMGPGYHRYIFGFSNNGIRLPGTEDRIVVYFTSLRGVRRTFEDCYAVRMIFRGFRVCIDERDVSMDSAYRKELQSVLGGKTVSLPQVFIRGNRVGDAEVIKQMFETGELAKVLDGFPRRQPGFVCEACGDVRFMPCGNCSGSRKVFDEDEGVPKRCLECNENGLIRCPDCGS*

>cassava4.1_024311m

MGCAASKPNTLHTNTNTNTNTNRLEATHSLHSPAFTSSSSSSHHYSSPPVTRAFSLQTPLVHHPPSKKGDSHHLVSLTSTTYGSLLLLEPISSKFNGENPPNQLHSPPEFTKNSHKTQPPADPGESFSPDSVINTWELMDGLDDDLGFEMGDSLKPKSSFSDHAIQVTSKSNSFQHLGFDSSAKKMNDSFDSVKSEEIAVDNSFSLSKPLWKHLSEESFLSKMDPNVVSSYRRALSSRQLGYTKESNGARSVGSSPMNSSSTHKNGFLSENTEDKIVLYFTSLRGIRKTYEDCCAVRMIFKGFRVPVDEKDISMDSSYRKELQSELKGKAMNLPQVFIKGKHIGGVEEIRQLNETGELAKVLEGFPVRDPRFVCESCGDARFLPCPNCHGSRKLFDEAEERLRRCLDCNENGLIRCPGCC*

>cassava4.1_011489m

MGCVSSNLLNNDDEFTQLGSSALSHHIVSLTSTTYGLLNLDPPPQSAATTPPTPPARFTLGSIFPSPLCEPKSLCPDPRPLRSDCPETIDSWELMSGLDNESFRFSPIIKKDRTASTEKENSNPNFTFNPTLESNILKPLKNSGFATSTPGSTPLKDNAHLLDRYEKLCPPNGENRVVIYTTTLRGIRKTFEACNFIRTAIEGFEVLICERDVSMDLGFREELRELMKGKEREATVPPRLFVKGRYVGGVEEVMKIVEEGKMGELLQGLPKKRAGDVCDGCGDVRFLPCFSCNGSSKIVMVVKEELGQKQGRTLVVRCPDCNENGLVLCPICA*

>cassava4.1_032796m

MQYQYQQFAESSSSWGFYVPPTAAIREMMGGSDALERVVRLASESAVVIFSMSSCCMCHTVKRLLCGMGVNPTVYELDQEPRGKDIERALMRLVGNYSNVVPVVFIGGKLIGAMDRVMASHINGTLVPLLKEAGALWL*

>cassava4.1_019777m

MAMTKAQDLVSSNSVVVFSKTYCPFCTTVKKLFDEVGAKYKTVELDTENDGSEIQAALGEWTGQKTVPNVFIGGKHIGGCDTTTGIHKEGKLIPLLTEAGAVAKASA*

>cassava4.1_018992m

MLKMPIKHHSREEASLFLLLLSLLLLKNAACASSSPSAFVQNVINSQRIVIFSKSYCPYCMRAKHIFSELHEQPYVVELDLRDDGAQIQYVLLDLVGRRTVPQVFVNGKHIGGSDDLNASAESGQLQKLLATD*

>cassava4.1_018177m

MQQAIPYKSWVPLYTTTTNPLTRPPPALTLSRTTANDNEQFLKGSKNMVSMVQENAIIVFARRGCCMSHVVKRLLLGLGVNPPIYEIDEEDEISVLQELEMIVNKIVGGGNDNVKKVQLPGVFIGGKLFGGLDRLMATHISGELVPILKEAGALWL*

>cassava4.1_024608m

MYQTESSWGSYMPPRNVGDPLERIERLASENAVVIFSISTCCMCHAIKRLFCGMGVHPTVHELDEDPRGKEMEKALMRLLGSSSAVPVVFIGGKLVGAMDRVMASHINGTLVPLLKEAGALWL*

>cassava4.1_017731m

MARPLSSILLKGIVGLPAVRSTRTVFGSLNCYGMRYSTTSPSDPDTHEDFRPNNKLEGSGIYLKDIVEQDVKDNPVMIYMKGVPDLPQCGFSSLAVRVLKHYNVPLSARNILEDPELKSAVKSFSNWPTFPQIFIKGEFIGGSDIIMNMHQSGELKEKLQDVAANQKSE*

>cassava4.1_024597m

MDRVRDLASTRAAVIFTKSSCCMCHSIKTLFYELGASPAIHELDHDANGREMEWALRGLGCNPSVPAVFIGGKYVGSAKDVLSLHLDGSLKQMLIDAKAIWF*

>cassava4.1_033785m

MDAVTRMVAERPLVIFSRSTCDMCHSIKTLILGFGANPTIYELDQIPNGQQIERALQQLGCQNLPAVFIGGECVGGDRQVMSLLLKNQLGPLLKRAGAIWVWND*

>cassava4.1_027058m

MDAVNELVKEKPVVIFSKSSCCMSHSIKSLICGFGANPTVYELDRIPNGEQIERTLVQQGCQPSVPAVFIGQKLIGGEKQVMSLHVQSQLIPLLINAGGIWI*

>cassava4.1_019956m

MEKVMGLASEKGVVIFSKSSCCMCYAVKILFQGIGVDPLVYEIDQDPEGREMERALMRMGCSAPVPAVFIGGKLIGSTNEIMSHHLSGTLIQMLKPYQTLS*

>cassava4.1_015641m

MWRQRSQSTVRIRDTSSSSSSTSSPFSFPTFKDIQHLCSDDPSPPTKNKTTPLQLPQPECQEPPPLISIPGADKRIVVYSTSLRIVRSTFEDCRTVQSILQGFRVSIDERDLSMDSRFLKELQQIFGGGQTTLTLPRVFIGGRYVGGAEEIRQLHEAGELKKFVEGLPAAEPGVCDTCSGYRFILCHDCNGSHKMYTEKSGFKSCAACNENGLIRCPSCSCAPL*

>cassava4.1_032411m

MGCASSKQKRCRRCRRSHVPHSPMPRSYSMHLVHPRQEKGNNYHLVALTSTTSSSLPLNSPSNCKNHINFAADILTAKGNDEENKNLRDGQLGISNNKELKSKEFSMGLIKAKAWSNTIQEKIPKIVPRTPVRTPPGEPETINAWELMAGLEEDDDSANKSNRFRSLSFDCSSDPAPVSDCPQLNGTTPNKSQTNCKSLWLQIADEEANSKSIPEFDPEIISTFRKALEDLSPTHPFHLNPPDSEKQPTPSPPSAGDNDIMKDFCKGENEKEKVVLYFTSLRGIRKTHEDCCHVRIILKGLGVRTDERDVSMHSGYKEELKELLGARFREGGLPKVFLGRKYIGGADEIRQLHEEGQLEKLLKGCGCEMAEAIGGGACEACGDVRFVPCETCFGSCKLYYERDDDLEEEEEEEEEEYDDDDYGFQRCPDCNENGLIRCPVCCY*

>cassava4.1_011619m

MRRASTLASSVFSRTLATTYDGGATTTNRRLLQAALYCTGGAGGSYNHNRWFYRLSNSFSARAASVGVAGALVSVAATTFLAQEAYAKEPPPQELVPKDVVLYQYEACPFCNKVKAFLDYYDIPYKVVEVNPISKKEIKWSDYKKVPILMVDGEQLVDSSVIIDKLRSKISKKKIDLASDGGGEEEKWRRWVDDHLVHVLSPNIYRNTSEALESFDYITSHGNFSFTEKFTVKYAGAAAMYFVSKKLKKKYNITDERAALYEAAETWVDALNGREFLGGSKPNLADLAVFGVLRPIRYLRSGRDMVEHTRIGEWYTRMEHAVGESSRIRA*

>cassava4.1_012950m

MVINQEPDIIDVAELMRGLEDDEMEIDDDICDKENIGPPIEVEDKLKTPFRRQTPLSEIDISSFRRPELSSGTILVPDLLAAFGQVVKEQVTMSEAESQTRTENENLERRETRRDLEKFEGEPPLKTRRIQDEDDDPLLSFEEKCPPGGNDSVILYTTTLRGIRKTFENCNSIRIILESFRVIFYERDVSMDKEFKEELWRVLESKVKPPRLFIKGRYIGGAEEILRLHEQGKFRILFKGVPIDHSTRPCEGCAGFRFVVCFHCRGSHRIVEDDGLSRRCHDCNENGLIICPLCC*

>cassava4.1_021286m

MDRVRDLASTRAAVIFTKSSCCMCHSIKTLFYELGASPAIHELDRDANGREMEWALRGLGCNPTVPAVFIGGKYVGSAKDVLSLHLDGSLKQKLIDAKAIWF*

>cassava4.1_019954m

MEKVMGLASEKGVVIFSKSSCCMCYAVKILFQGIGVEPLVYEIDQDTEGREMERALMRLGCSAPVPAVFIGGKHMGSTNEIMSLHLSGTLIQMLKPYRALS*

>cassava4.1_025892m

MDAVTRLVAERPLVIFSKSTCDMCHSIKTLVRGFGANPTVYELDQIPNGQQIERALQQLGCQNLPAVFIGGDYVGGDRQVMSLLLKNQLGALLKRAGAIWVWNDS*

>cassava4.1_029365m

MRRVNGLVTVIRTVAAASAAGTTARHRVFQAAMMSTCASTNSSQRIADRFGISSPSVAHGVAGTMFFSVAASSLEQEVHAKEAPPAEKFLPKDVVLYQYEACPFCNKVKAFLDYYSIPYKIVEVNPISKKEIKWSNYKKVPILTVDGEQMVDSSDIINKLFQRIIQMIQSQVMMKKANGRVHVLSPNIYRTTSEALESFDYITTHGNFSFTERLVAKYAGAAAMYFVSKKLKKRHNITDERAALYEAAETWVNALKGREYLGGSKPNLADLAVFGVLRPIRHLKSGKDMVEHTHIGEWYSRMEQAVGEPARIKA*

>cassava4.1_034271m

MKSMKGRFLKKLRFIPTIHTLKHGLVSHLNSTDKFSNQILQIPSVYIQEDHKINNLQEPVTADIAVSELKNEESDNLELHVHDDEKITPFTVFNDNVPAAIDNLESPVSSEITMEYDTADEADHEKYTEEYSSLSHFEEKCPPEGSESIVLYTTSLRSIRKTFEDCHTILFLLESFKVEFYERDVSMHLEYREELWRILGGRVIPPRLFIKGRYIGGADEVVSLHEQGKLKKLLEGIPKLLSNAPCVGCGNKRFVVCFNCNGSRRVFNGGESDEMNIIRCSECNENGLVKCSICYR*

>cassava4.1_025754m

MGCVSSKPFRKQLHRDIIIDNDGGASVNHVVSLTSTTYGALKLDINEKRQQQQEHEQEPIKEIVAESKKLQQRSPPREEPEIINAWELMKDLEDGGLPISNHPKKSPKMRALRREIADMDARSPLKFMNQIGSPRKAKTFGGKENKVKKVSEFSPRAVLKAKNSSGKSSKAMLRLSYPVKSSPIWTKTENAESFSPLFDPELVELYERELSEEEGKIKKIISTTPRPQKSKNARELESILHSFEQKCPTGGENEVVIYTTTLRGIRKTFEECNTVRSIIESYHIHMLERDVSMDSGFKEEIRELMGTKEVKLPLVFVKGRFIGGADQVVKLEEEGKLEILFDGVPRGLASDCHGCAGLRFMMCVKCNGSCKVLDEKHKKMVKCGECNENGLRHCPICC*

>cassava4.1_012569m

MASISLSPLQTSPSLRVLSSYTPQTAPNLSFYSHSKPSIPFPSTSLKPYTPIIPRSRSRSRSRSLIVPAAVKKISETDLVAVPLTAEEFSQKLPSESGVYAVYDKNDDLQFIGISRNVGASVFSHLKSVPDLCSSVKVGIVDEPDKESLTQAWKSWMEEHIKTTGKVPPGNQSGNATWIRQPPKKKSDLRLTPGRHVQLTVPLEELIGKLVKESQVVAFIKGSRNAPLCGFSQRVIGILETQGIDYESVDVLDEEYNYGLRETLKKYSNWPTFPQIFVNGELIGGCDILTSMHEKGELAGLLKK*

>AT2G47880.1

MDKVMRMSSEKGVVIFTKSSCCLCYAVQILFRDLRVQPTIHEIDNDPDCREIEKALLRLGCSTAVPAVFVGGKLVGSTNEVMSLHLSGSLVPLIKPYQSILY*

>AT2G38270.1

MAAITISSSLHASASPRVVRPHVSRNTPVITLYSRFTPSFSFPSLSFTLRDTAPSRRRSFFIASAVKSLTETELLPITEADSIPSASGVYAVYDKSDELQFVGISRNIAASVSAHLKSVPELCGSVKVGIVEEPDKAVLTQAWKLWIEEHIKVTGKVPPGNKSGNNTFVKQTPRKKSDIRLTPGRHVELTVPLEELIDRLVKESKVVAFIKGSRSAPQCGFSQRVVGILESQGVDYETVDVLDDEYNHGLRETLKNYSNWPTFPQIFVKGELVGGCDILTSMYENGELANILN*

>AT2G30540.1

MDKVVRMSSEKGVVIFSKSSCCMSYAVQVLFQDLGVHPTVHEIDKDPECREIEKALMRLGCSTPVPAIFVGGKLIGSTNEVMSLHLSGSLVPLVKPFQANLC*

>AT2G47870.1

MERVRDLASEKAAVIFTKSSCCMCHSIKTLFYELGASPAIHELDKDPQGPDMERALFRVFGSNPAVPAVFVGGRYVGSAKDVISFHVDGSLKQMLKASNAIWL*

>AT2G20270.2

MVAATVNLANMTWTSLNSNPAISFSMLSGIRNLGMLPFRRCLKPTVIGIASWPPLRCSSVKAMSSSSSSSGSTLEETVKTTVAENPVVVYSKTWCSYSSQVKSLFKSLQVEPLVVELDQLVSLGKTSLPHDIGLKHLQKFWWFLAFPGSEGSQLQNVLEKITGQYTVPNVFIGGKHIGGCSDTLQLHNKGELEAILAEANGKNGQT*

>AT2G41330.1

MGSSVSKTASSASASSITSILNSPPETEFNIPSSSVPKAFTFPMPSIHHPPAKKGDTHHFVSLTSTTYGSLVLDDRQTLPHISVSGKSNKKMPETEEARDSFSPDSVINTWELMNDLDDEFDSANSDTSKSNSVVNLDSFSKPITNRDVVINGSAYGSYEDEEDWRLLPFKPKQPLWKHMSEESFLSDLDPNIISSYKRALSSKQLGKNSSNGHSNHSVLVSLPEYVSSSPLSSQTSEKDQEKPRLLEKEDNENKIVVYFTSLRGIRKTYEDCCCVRAILRGFQVAVEERDISMDSKYRKELQNALGEEKPVCLPQVFIRGVRIGGIEEIKILNDGGELAEMLKDFPACESIGACDSCGDARFVPCTNCGGSTKVFEEQEDGFKRCNGCNENGLVRCNKCCL*

>AT4G33040.1

MMQELGLQRFSNDVVRLDLTPPSQTSSTSLSIDEEESTEAKIRRLISEHPVIIFSRSSCCMCHVMKRLLATIGVIPTVIELDDHEVSSLPTALQDEYSGGVSVVGPPPAVFIGRECVGGLESLVALHLSGQLVPKLVQVGALWV*

>AT4G10630.1

MGCVSSNLLNHDEDFSQIGGGSSAFGHHIVKLTSTTYGLLTLDPPPPPSPPMTPPEKFTVDTKSKSIWSEPRVIKSEPEIINSWELMSGLDGESFRFTPLPKTPVKYKVFGGENKENSDPSRRNPRKNLNDEVLKPLDLNREDSDSNSRSPRKSFKPLDLKLDEKFERICPPGGENRVVMYTTSLRGVRQTFEACNAVRAAVESFGVVVCERDVSMDRRFREELVSLMAKRVGDEGVAALPPRVFVKGRYIGGGEEVLRLVEEGSFGELISGIPRKKAGGCESGACDGCGGLFFLPCFRCNGSCKMVKGWGSASVVVRCNECNENGLVPCPICS*

>AT4G15690.1

MENLQKMISEKSVVIFSKNSCCMSHTIKTLFLDFGVNPTIYELDEINIGREIEQALAQLGCSPTVPVVFIGGQLVGGANQVMSLHLNRSLVPMLKRAGALWL*

>AT4G04950.1

MSGTVKDIVSKAELDNLRQSGAPVVLHFWASWCDASKQMDQVFSHLATDFPRAHFFRVEAEEHPEISEAYSVAAVPYFVFFKDGKTVDTLEGADPSSLANKVGKVAGSSTSAEPAAPASLGLAAGPTILETVKENAKASLQDRAQPVSTADALKSRLEKLTNSHPVMLFMKGIPEEPRCGFSRKVVDILKEVNVDFGSFDILSDNEVREGLKKFSNWPTFPQLYCNGELLGGADIAIAMHESGELKDAFKDLGITTVGSKESQDEAGKGGGVSSGNTGLSETLRARLEGLVNSKPVMLFMKGRPEEPKCGFSGKVVEILNQEKIEFGSFDILLDDEVRQGLKVYSNWSSYPQLYVKGELMGGSDIVLEMQKSGELKKVLTEKGITGEQSLEDRLKALINSSEVMLFMKGSPDEPKCGFSSKVVKALRGENVSFGSFDILTDEEVRQGIKNFSNWPTFPQLYYKGELIGGCDIIMELSESGDLKATLSE*

>AT4G15680.1

MDKLQKMISEKSVVIFSKNSCCMSHTIKTLFIDFGVNPTIYELDEINRGKEIEQALAQLGCSPTVPVVFIGGQLVGGANQVMSLHLNRSLVPMLKRVGALWL*

>AT4G15670.1

MEKLQKMTSEKSLVIFSKNSCCMSHTIKTLFLDLGVNPTIYELDEINRGKEIEQALAQLGCSPTVPVVFIGGQLVGGANQVMSLHLNRSLIPMLKRVGALWL*

>AT4G28730.1

MAVTAFNTLKLVSSSLDPIPSVSCSSYSFSLIYVGSPYKRCLKQSCSVRAMTSSSSAASSSSSSFGSRMEESIRKTVTENTVVIYSKTWCSYCTEVKTLFKRLGVQPLVVELDQLGPQGPQLQKVLERLTGQHTVPNVFVCGKHIGGCTDTVKLNRKGDLELMLAEANGKNGQS*

>AT4G08550.1

MESSPKQQNTEEERKSAEIIAKEVSPKQQNVEEERKSAEIIAKEVSPKHNVEKKEEEFTRKPVVEIEEEEEEMESIDIHEEEEGDNNVSLDEIMSVDSSDDDDDSESSAEITKACEETVVDERSGISQESNSSTSSAWTEKAAAIKNFVRAKSEVAVHTMIRRLSGKLSIDNAAHGTKDDEVESPKTEGKSLWNPLSYLKMMQNDEDLVYREEETVFEPVVMKGRIILYTRLGCEECRGCRLFLHEKRLRYVEINIDIYPTRKVELEKISGGDVVPMVFFNEKLVGSYKELKVLEESGELEEKIKHLIEETPPREAPLPPFSGEDDASSKGPVDELALIVLKMKPCVVKDRFYKMRRFKNCFLGSEAVDFLSADQRLERDGPRPIVEIASRLRLVYRAILEAYTSPDGKHVDYRSIHGSEEFARYLRIIQELHRVELEDMQREEKLAFFINLYNMMAIHSILVWGHPAGTFDRTKMFMDFKYVIGGYTYSLSAIQNGILRGNQRPMFNPMKPFGVKDKRSKVALPYAEPLTHFTLVCGTRSGPPLRCFTPGEIDKELMEAARDFLRCGGLRVDLNAKVAEISKIFDWYGVDFGNGKEEILKHASTFLEPQLSEALLDCLVDTQFEVKYQPYDWGLNN*

>AT4G15700.1

MENLQKMISEKSVVIFSKNSCCMSHTIKTLFLDLGVNPTIYELDEISRGKEIEHALAQLGCSPTVPVVFIGGQLVGGANQVMSLHLNRSLVPMLKRAGALWL*

>AT4G15660.1

MEKIQKMISEKSVVIFSNNSCCMSHTIKTLFLDLGVNPTIYELDEINRGKEIEYALAQLGCSPTVPVVFIGGQLVGGANQVMSLHLNRSLIPMLKRFGALWL*

>AT1G77370.1

MVDQSPRRVVVAALLLFVVLCDLSNSAGAANSVSAFVQNAILSNKIVIFSKSYCPYCLRSKRIFSQLKEEPFVVELDQREDGDQIQYELLEFVGRRTVPQVFVNGKHIGGSDDLGAALESGQLQKLLAAS*

>AT1G28480.1

MQGTISCARNYNMTTTVGESLRPLSLKTQGNGERVRMVVEENAVIVIGRRGCCMCHVVRRLLLGLGVNPAVLEIDEEREDEVLSELENIGVQGGGGTVKLPAVYVGGRLFGGLDRVMATHISGELVPILKEVGALWL*

>AT1G06830.1

MDKVMRMSSEKGVVIFTKSSCCLSYAVQVLFQDLGVNPKIHEIDKDPECREIEKALMRLGCSKPVPAVFIGGKLVGSTNEVMSMHLSSSLVPLVKPYLC*

>AT1G03020.1

MEKISNLLEDKPVVIFSKTSCCMSHSIKSLISGYGANSTVYELDEMSNGPEIERALVELGCKPTVPAVFIGQELVGGANQLMSLQVRNQLASLLRRAGAIWI*

>AT1G03850.1

MQKAIRPYESPWTKTVPGNSIFLLKNEDKPSSSSSSLSWLTSGSPKPTSISNKRSSNLVVMENAVVVFARRGCCLGHVAKRLLLTHGVNPVVVEIGEEDNNNYDNIVSDKEKLPMMYIGGKLFGGLENLMAAHINGHSIKIRTDTWSSFSVATVDRIRW*

>AT1G64500.1

MGCTSSKQAKANVVADVYKPPPSSFAVFDVNAIQEPWLKFEHEDDEKPPRSTVFDRLDEDDDDDDDDGDDAPKTWEEVSKSLETKLKPAAVKPPEVDSVKPPATPPRRLPRKSASFHTLDELEVRAKRSIAAQIPTTMVKLKRTESMSKLRPESDDRTESTQSSYSGPRSVKENIFVKRDRERREKEGNKKPVMNWDPLREFPEKCPPGGGEGLIVYTTSLQGVRRTYEDCMRVRAIMEQQGVVVDERDVSLDAGVLSELKELLQDEASVAPPRVFVKGRYLGGAAEVTAMNENGKLGRVLRWARVERVGEEGRLTCEGCGGARWLPCFECGGSCKVAAVGAAKGERWERCVKCNENGLIRCPVCFVN*

>AT1G32760.1

MGGCVSSNLLTPTDDSSFSHLTSSSLSQHFVKLTSTTYGLLNLDSSSPSPPSPSSSSSAKSVITPMTPPERFTINGKEAAMMMVKSEPTTEVINFWELMSGLDGDTCRFSPIPVKCNGFSGGLKKENSDPNLKNPNDYEVLKPLDPKLAEESERLCDGGENRVVIYTTSLRGVRRTFEACNAVRAAIESFGVVVCERDVSMDRGFREELSNLMAVESTAAVLPPRVFVKGKYIGGAEEVMRLVEEGLLGELLKEIPRKKDRCGGGCGGCGGLAFLPCSGCNGSCKVVEGWGNDAVVVKCKECNENGLVRCPICS*

>AT3G54900.1

MALRSVKTPTLITSVAVVSSSVTNKPHSIRFSLKPTSALVVHNHQLSFYGSNLKLKPTKFRCSASALTPQLKDTLEKLVNSEKVVLFMKGTRDFPMCGFSNTVVQILKNLNVPFEDVNILENEMLRQGLKEYSNWPTFPQLYIGGEFFGGCDITLEAFKTGELQEEVEKAMCS*

>AT3G28850.1

MGCASSKHRKRCLHCRRGYSPPVDVQRSHSVHHASQNSEDSCHMVALSSSSLGSLKLCDSSFGHNHKHLADFSEKLVSGESVKTGNGFGPNVVREKSDKEKSNLELQAKLMEAKVWSSMMNEKIPKIVPKTPIVTPPGEPETINTWEMMDGLEDVLSPLRSPNHVKSFSFDVGPNGGKSNGSVKPVWLQMEEEEEGFEDFDPEIISSFRKSLQELPSDHPFHISNHDFELKPRFNFSDEEKEEEEQSVGKERVILYFTSLRGIRKTYEESCDVRVILKSLGIRVDERDVSMHSGFKDELKELLGEKFNKGVGITLPRVFLGRKYIGGAEEIRKLNEDGKLEKLLGGCERVEENQNGNGLECEACGDVRFVPCETCSGSCKVYYEYEDDDDDDDEGDDDESVKEEREYGFQTCPDCNENGLIRCPVCCD*

>AT3G62930.1

MESVRSLVEDKPVVIFSKSSCCMSHSIQTLISGFGAKMTVYELDQFSNGQEIEKALVQMGCKPSVPAVFIGQQFIGGANQVMTLQVKNQLAAMLRRAGAIWV*

>AT3G11920.1

MRFEESKNNCQNPRKSEKVTNGKSYTISSDIVSPPYSNSNPISDHVHPATPIPEAVSQSPMKEISIDDPYLPPHPQLPKLEPPRGRRVSLEELLHPKEKFTGDLMKFIRQSGNAISKRISVLLENDDDDDSIDDVTEFKISGVKVLVKMKNEEEIKGRITFFSRSNCRDSTAVRLFLRERGFDFSEINIDVYSSREKELVERTGSSQVPQIFFNEKHFGGLMALNSLRNSGEFDRRVKEFLKEKCCGDAPLPVMYGFDEESNNKDVVVVVDEMMRFVRVLRQKLPIKDRLMKMKIVKNCFSGAEMVEILIDYLDCGRKKAVEIGKRLAEKHFIHHVFGENEFEDGNHYYRFLEHEPFVSKCYNFRGSTNDMEPQSAAIVGQKLFKIMTAILESYSSNDHTSVDYMRISQSEEFRRYLNLAQDFHRLNLVELSTEEKLAFFLNLYNAMVIHALISIGRPEGLIARRSFFTDFQYVVGGYSYSLSSIRNDILRRGRKPSYPFIRPPFNNGKTRHELGLLKLNPLVHFGLCDGTKSSPVVRFFTPQGVEAELKRAAREFFQNGGIEVVLDKRTIHLSRIIKWYKEDFSEEKKMLKWIMGYIDSNDAGLLTHLLGDGGGSFNIVYQDYDWSTNN*

>AT3G62960.1

MDKVMRMSSEKGVVIFTKSSCCLCYAVQILFRDLRVQPTIHEIDNDPDCREIEKALVRLGCANAVPAVFVSGKLVGSTNDVMSLHLSGSLVPLIKPYQSFHN*

>AT3G02000.1

MQYQTESWGSYKMSSLGFGGLGMVADTGLLRIESLASESAVVIFSVSTCCMCHAVKGLFRGMGVSPAVHELDLHPYGGDIQRALIRLLGCSGSSSPGSLPVVFIGGKLVGAMDRVMASHINGSLVPLLKDAGALWL*

>AT3G21460.1

MDVVARLASQRAVVIFSKSTCCMSHAIKRLFYEQGVSPAIVEIDQDMYGKDIEWALARLGCSPTVPAVFVGGKFVGTANTVMTLHLNGSLKILLKEAGALWL*

>AT3G15660.1

MAASLSSRLIKGIANLKAVRSSRLTSASVYQNGMMRFSSTVPSDSDTHDDFKPTQKVPPDSTDSLKDIVENDVKDNPVMIYMKGVPESPQCGFSSLAVRVLQQYNVPISSRNILEDQELKNAVKSFSHWPTFPQIFIKGEFIGGSDIILNMHKEGELEQKLKDVSGNQD*

>AT3G11773.2

MMDMKERMVKKLKLITSLGNLKQAIALHKSNLDHTIFHQIDEKECLTPTTIEVSEDKRLQQNLDELLWAFEEKCVAGGQDSVASSQQASFRERDVSMDCEYKEEMWRLLGEQVTPPRLFIKCKYIGGADEVVSLNENEKLKKLLEVFSSAKSRQCEMCENERFLICSKCNGRSRVVAEHETWKRCIECNENGLVKCALCT*

>AT3G62950.1

MERIRDLSSKKAAVIFTKSSCCMCHSIKTLFYELGASPAIHELDKDPEGREMERALRALGSSNPAVPAVFVGGRYIGSAKDIISFHVDGSLKQMLKDAKAIWL*

>AT3G57070.1

MGSSASKTASSVSSASSSSAFSPPPTKFSSDSSSPAVQRAFSFPTPLVHHPPARKGDSHHLVSLTSTSYGSLLLLDLDGSKNSSSDQQTLPRISISGKNTPDPVSPDSVINTWELMDGLDDEFEFEIPKPGKRLNSDFCSKPDPNRNVSLNGSSLKLDESYEIVRIEEDDGDWVPLTYKPKQPLWKHLSEESFLSDLDPSIVSSYKKALSSKLLSNHSNTRNPHRPTKSLSCSPSSNPSILISEEPKSVSSSQLISSPAKPRLPGTEDKIVLYFTTLRGIRKTYEDCCCVRAILRGVQVSVDERDISMDSKYRKELQSVLGAAEKPVCLPQVFIRGTHIGGVEEIMQLNDGGELAEMLKDFPACERLGTCRSCGDARFVPCTNCDGSTKVFEEQDERFKRCPKCNENGLVRCRVCCL*

>AT5G14070.1

MQYKTETRGSLSYNNNSKVMNNMNVFPSETLAKIESMAAENAVVIFSVSTCCMCHAIKRLFRGMGVSPAVHELDLLPYGVEIHRALLRLLGCSSGGATSPGALPVVFIGGKMVGAMERVMASHINGSLVPLLKDAGALWL*

>AT5G03870.1

MGCVSSKLGKKKLIREIRVNNGGDHIVSLTSTTYGHLDLDERAETSPKSLEVTKGEVFESEIIPRRSIKRDDPEIINTWELMEDLEDSMHVSNPQKISPKSRGIFGKSWKTPVKSVVESPKRGSSKRFGGKENNSRGVSPNQILKPKNILETPKRGVMRLSFPLKSEESSVTVTQRRKSYSPMFDPDLVASYERELSQEKEQIKMVISPVVHESRKTEKTERILEKFPEKCPPGGEHSVVIYITTLRGIRKTFEDCNVVRSILDSHEVRFSERDVSMHSVFKEEIRGIMGTKHVKIPAVFVKGRMVGSVEEVMRLEEEGKLGILLEGIPAARLGGSCCRGCGGMRFMMCVVCNGSCKVREEEKKSMVKCLKCNENGLVLCPICS*

>AT5G11930.1

MKTMRGLRNCSNDAVTLDLTVHPPPPPPLPPPAPSTVSSSTASTSLSFDEEETSESKIGRLISEHPVIIFTRFSSCCMCHVMKKLLSTVGVHPTVIEIDDGEIAYLAVEAAPVLFIGGTCVGGFESLVALHLSGQLIPRLVEVGALWA*

>AT5G18600.1

MDMITKMVMERPVVIYSKSSCCMSHTIKTLLCDFGANPAVYELDEISRGREIEQALLRLGCSPAVPGVFIGGELVGGANEVMSLHLNGSLIPMLKRAGALWV*

>AT5G39865.1

MGCASSKNRNRCRNCKGGLSPVIVPRSYSMHVHHPAQHTGDSYHTVALTSSTIGSLSLCDSSLRHFHKHLEDSFYKQRVSDQMGEETLISGNGFLHGDEEKMNLDLQAKVIEAKVWSSTINEKIPKIVAKTPIVTPPGEPETINTWELMEGLEDVSPLRSPNHLRSFSFDFVRIQPSHDHDHDVAVSFDLPKSRFHENVKSSCRVDDLDPPDIVSRFKRKTLGKERVVLYFTSLRGIRKTYEDCCNIRIILKSLGIRIDERDVSMHSGFKDELKKLLEGKFNNGVGITLPRVFLGNKYLGGVEEIKKLNENGELEKLIKDCEMVEDGSPGFGNECEACGDVRFVPCETCSGSCKLYHEGEEEDEGVTEYGFQRCPYCNENGLIRCHVCCE*

>AT5G58530.1

MWRPWRKSSVKIHDTYSPTTASFKDIHHLCSDDSPSFPSSPSPSPKTASRVFHRVRAANLILKSWPTRQSNHLLRADSEPINLSRNQNPESDSKQSKTKTEPDVRISIPGAEKSIVVYFTSLRVVRPTFEACKSVTSILHSFPVRIDERDLSMDASFSTELQRIFGKDQNQNQNQAKTPKLPRVFIGGRYIGGAEEVKQLHEIGELKKLVQELPKIEPGVCEMCGGHRFVPCKDCHGSHKVHTEKLGFRTCLTCNENGLVRCSSCSFPQSPPP*

>AT5G20500.1

MTMFRSISMVMLLVALVTFISMVSSAASSPEADFVKKTISSHKIVIFSKSYCPYCKKAKSVFRELDQVPYVVELDEREDGWSIQTALGEIVGRRTVPQVFINGKHLGGSDDTVDAYESGELAKLLGVSGNKEAEL*

>AT5G40370.2

MDSIMCVSSWKFYTMNDSWMYLDAFVYQWMILSYLIGNFSGICSKTYCPYCVRVKELLQQLGAKFKAVELDTESDGSQIQSGLAEWTGQRTVPNVFIGGNHIGGCDATSNLHKDGKLVPLLTEAGAIAGKTATTSA*

>AT5G13810.1

MAGLEKNADFSGKTTKSATTTSFFNRSLTIHGRTVVDSGPKSHNLNPSLNRTTSITKFYTPVESMGTSLKGKVKNLCRLFETSKPVKPALAEIPQKQKSGKSLLPESRISPFSSLNNSVIRLPGTEDRIVVYFTSLRGIRRTYEDCYAVRMIFRGFRVWIDERDVSMDIAYRKELQIAMGEKSVSLPQVFIMGKYVGGADVIKSLFEIGELAKILKEFPMRQPGFVCHCCGDIRFVPCSNCSGSKKLFDEDEDRVKRCPECNENGLIRCPDCSS*

>AT5G63030.1

MGSMFSGNRMSKEEMEVVVNKAKEIVSAYPVVVFSKTYCGYCQRVKQLLTQLGATFKVLELDEMSDGGEIQSALSEWTGQTTVPNVFIKGNHIGGCDRVMETNKQGKLVPLLTEAGAIADNSSQL*

>AT5G42150.1

MRRVTGLAARTISSSVAINSRLTQSMAITTISSSEPISRRFGGLPEIKTPSFAGGVAGVVFFSAAAVSSLGQEVHAKEMAQKFNPKEVVLYQYEACPFCNKVKAFLDYNKIPYKVVEVNPISKKEIKWSDYKKVPILTVDGEQMVDSSVIIDSLFQKMHPEISKSEDDEETKWRKWVDNHLVHLLSPNIYRNTSEALESFEYITTHGNFSFTERLVAKYAGATAMYFVSKKLKKKYNITDERAALYDAAETWVDALKERPYLGGSKPNLGDLAVFGVLRPIRYLRSGKDMVDNTRIGEWYSRMENTVGEPSRIKE*

>AT5G01420.1

MKSSRMKFAKKLKSIRAGEYLNQDRILQVLSAADEFLPKISNHIPQITSAASIWRTPQKSENQIANVTELLENVRQGEEEASGNFVGDEENVRPPVNQIPRVPCDRKESKLAVSCGSDTGIGRKSFGSGFRRPDLNSTTLFDPKLLEAFELAALCFRKIKDFSREARVNDEDDIVFPEEEEIRKDGNSIPPVISVVEYRRKDENVVVLHKDDGNALQIVNAIREEVIGEEDIGKDGNTLMDPLLEFEERCPPGGEESVVFYTTTLRGIRKTFDDCNMIRFLLDSFKVKYYERDVSMHREYREELRRISAAETEVLPPVLFVKGRCIGGAQRVLGLHEQGKFKILFEGIPITGDERCRRCDGFRFLMCDGCRGSRRIISGDGSRIQCLICNENGLIVCVGCS*

>AT5G06470.1

MKGMKERLVKKIKLITTVKTLKQSLTLHKSTIDRTIHHLRDEIVAESGQSKGCVTPTLLELEDAEEHNLEEERGILLEFKENCPPGGEDSVVFYTTGLRSVRKTFEACRRVRFLLENHQVMYRERDVSMDSEFREEMWRLLGGKVTSPRLFIRGRYIGGAEEVVALNENGKLKKLLQGISQVDSPCESCENERFLICSSCNGSTRLLAEHHDEESSNDNMWTRCRECNENGLVKCPLCT*
