# Supplementary material for: Identification and characterization of drought-responsive CC-type glutaredoxins from cassava cultivars reveals their involvement in ABA signalling
Source: BMC Plant Biol. 2018 Dec 4;18:329. doi: 10.1186/s12870-018-1528-6 (PMC6280520; doi:10.1186/s12870-018-1528-6)
Supplement: Supplementary file 3 — Table S6. Protein sequences of TGA transcription factors from cassava. and Arabidopsis. (DOC 35 kb) [file 12870_2018_1528_MOESM3_ESM.doc]

Identification and characterization of drought-responsive CC-type glutaredoxins from cassava cultivars reveals their involvement in ABA signalling.

Meng-Bin Ruan^1,2,†,*^, Yi-Ling Yang^3,†^, Xin Guo^4^, Bin Wang^4^, Xiao-Ling Yu^1,2^, Kai-Mian Li ^5^, and Ming Peng^1,2,*^

Table S6. Protein sequences of TGA transcription factors from cassava and Arabidopsis.

>MeTGA074

MGSRVGLEDEKEANGMLSFDPQLPISNALGIEGSTIHPYRVSGFGLFEQSVAFHFEDVVDLSTNTVFNSAKASSQEVSSDPLNIGTSDKLTTSLNINPSAAQVESQRLPPEKKQQLNLVSISSGNTENWGESNMADASPRTDISTDGDTDDKNPRFAYGQSNAVAVSDSSDRSKDKLDQKTLRRLAQNREAARKSRLRKKAYVQQLESSRLKLTQLEQELQRARQQGVFISSSGDQAHSMSGNGAMAFDVEYARWLEEQNRQINELRSAVNTHAGDTELRIIIDGIMAHYDEIFRLKGNAAAADVFHLLSGMWKTPAERCFLWLGGFRSSELLKLLVNQLEPLTEQQLVGIGNLQQSSQQAEDALSQGMEALQQSLAETLSSGSLGSSSSSGNVANYMGQMAMAMGKLGTLEGFIRQADNLRQQTLQQMHRILTTRQSARALLAIHDYFSRLRALSSLWLARP

>MeTGA304

MASHGVGETGLSDSGPSNHHLPYAALHGINAPSTSFFNQEGSPFDFGELEEAVLQGVKIRNDEAKAPLFRPAATLEMFPPWPIRFQQTPRGSSKSGGESTDSGSAVNTLSSKAEAQLDSDSPISKKASSSDHHQAFDQKHLQLQQHQQQMEMASNTSRTGAPSELNPSPAKLPQEKRKGSTSEKQLDAKTLRRLAQNREAARKSRLRKKAYVQQLESSRIKLTQLEQDLQRARQQGLFLGGCSGVGGNISPGAAIFDMEYARWLEDDHRHMSELRTGLQAHLSDGDLRVIVDRYISHYDEIFRLKGVAAKSDVFHLVTGMWSTPAERCFLWMGGFRPSELIKMLTSQLDPLTDQQIVGICSLQHSSQQAEEALSQGLEQLQQSLVDTIASGQIVDGMQQMAVALGKLANLEGFVRQADNLRQQTLHQLRRILTVRQAARCFLVIGEYYGRLRALSSLWASRPRESLMGEENACQTGSDLQMVEPPPNHFSNF*

>MeTGA351

MQSFKHFPLPEMYCHSSFFLRGEDGSRNQTRFADLGELEQPAPAFHHDDAVDLSPSSMFSLKSGNVAVLSSNLQYDAVLNTSIGSAEIATTGTGCLDTGQYMYHKGTTIASSLGNAHCIENWGDSGMADNSQQTDTSTDVDTDDRNQLHGVQHGTVMVVDSMEKSKAKAGDQKTLRRLAQNREAARKSRLRKKAYVQQLESSRHRLAQLEQELQRARQQGIFVASGLSGDHGAGNGAVAFNMDYARWLEEHQRLISDLRSAVNSHMGDNELHVLVDAVMSHYDEIFRLKSIGTKADVFHMLSGMWKTPAERCFMWLGGFRSSELLKILGNHLEPLTDQQLMGICNLQQSSQQAEDALSQGMEALQQSLVETVSSTSLGPAGSGKVADYMGQMAIAMGKLATLENFIHQADLLRQQTLQQMNRILTTRQAARALLVISDYTSRLRALSSLWLARPRD*

>MeTGA813

MNSPSTQFVSPGRMGMYEPIHQIGMWGENFKSNGISNASPSMFIPGNPNSSQSILIPAETKLDNQSEDTSHGTLGPSSSKYDQEASKPIDKVQRRLAQNREAARKSRLRKKAYVQQLESSRLKLFQLEQELERARHQGLYVGGLETSQMGFAGPINPGIANFEMEYGHWLEEQNKNIHDLRNALNAHISDNELRILVDTGINHYSELFRMKATAAKADVFYLMSGMWKSSAERFFLWIGGFRPSELLKVLKPQLEPLTDQQLLDVSNLKQSCQQAEDALSQGMEKLQQTLAETVAAGRLGEASHMPQMDTAMEKLEVGRNQLPL

>PAN

MQSSFKTVPFTPDFYSQSSYFFRGDSCLEEFHQPVNGFHHEEAIDLSPNVTIASANLHYTTFDTVMDCGGGGGGGLRERLEGGEEECLDTGQLVYQKGTRLVGGGVGEVNSSWCDSVSAMADNSQHTDTSTDIDTDDKTQLNGGHQGMLLATNCSDQSNVKSSDQRTLRRLAQNREAARKSRLRKKAYVQQLENSRIRLAQLEEELKRARQQGSLVERGVSADHTHLAAGNGVFSFELEYTRWKEEHQRMINDLRSGVNSQLGDNDLRVLVDAVMSHYDEIFRLKGIGTKVDVFHMLSGMWKTPAERFFMWLGGFRSSELLKILGNHVDPLTDQQLIGICNLQQSSQQAEDALSQGMEALQQSLLETLSSASMGPNSSANVADYMGHMAMAMGKLGTLENFLRQADLLRQQTLQQLHRILTTRQAARAFLVIHDYISRLRALSSLWLARPRD

>AtTGA1

MFDQEASTSRHPDKIQRRLAQNREAARKSRLRKKAYVQQLETSRLKLIQLEQELDRARQQGFYVGNGIDTNSLGFSETMNPGIAAFEMEYGHWVEEQNRQICELRTVLHGHINDIELRSLVENAMKHYFELFRMKSSAAKADVFFVMSGMWRTSAERFFLWIGGFRPSDLLKVLLPHFDVLTDQQLLDVCNLKQSCQQAEDALTQGMEKLQHTLADCVAAGQLGEGSYIPQVNSAMDRLEALVSFVNQADHLRHETLQQMYRILTTRQAARGLLALGEYFQRLRALSSSWATRHREPT

>AtTGA2

MADTSPRTDVSTDDDTDHPDLGSEGALVNTAASDSSDRSKGKMDQKTLRRLAQNREAARKSRLRKKAYVQQLENSRLKLTQLEQELQRARQQGVFISGTGDQAHSTGGNGALAFDAEHSRWLEEKNKQMNELRSALNAHAGDSELRIIVDGVMAHYEELFRIKSNAAKNDVFHLLSGMWKTPAERCFLWLGGFRSSELLKLLANQLEPMTERQLMGINNLQQTSQQAEDALSQGMESLQQSLADTLSSGTLGSSSSGNVASYMGQMAMAMGKLGTLEGFIRQADNLRLQTLQQMIRVLTTRQSARALLAIHDYFSRLRALSSLWLARPRE

>AtTGA3

MEMMSSSSSTTQVVSFRDMGMYEPFQQLSGWESPFKSDINNITSNQNNNQSSSTTLEVDARPEADDNNRVNYTSVYNNSLEAEPSSNNDQDEDRINDKMKRRLAQNREAARKSRLRKKAHVQQLEESRLKLSQLEQELVRARQQGLCVRNSSDTSYLGPAGNMNSGIAAFEMEYTHWLEEQNRRVSEIRTALQAHIGDIELKMLVDSCLNHYANLFRMKADAAKADVFFLMSGMWRTSTERFFQWIGGFRPSELLNVVMPYVEPLTDQQLLEVRNLQQSSQQAEEALSQGLDKLQQGLVESIAIQIKVVESVNHGAPMASAMENLQALESFVNQADHLRQQTLQQMSKILTTRQAARGLLALGEYFHRLRALSSLWAARPREHT

>AtTGA4

MNTTSTHFVPPRRFEVYEPLNQIGMWEESFKNNGDMYTPGSIIIPTNEKPDSLSEDTSHGTEGTPHKFDQEASTSRHPDKIQRRLAQNREAARKSRLRKKAYVQQLETSRLKLIHLEQELDRARQQGFYVGNGVDTNALSFSDNMSSGIVAFEMEYGHWVEEQNRQICELRTVLHGQVSDIELRSLVENAMKHYFQLFRMKSAAAKIDVFYVMSGMWKTSAERFFLWIGGFRPSELLKVLLPHFDPLTDQQLLDVCNLRQSCQQAEDALSQGMEKLQHTLAESVAAGKLGEGSYIPQMTCAMERLEALVSFVNQADHLRHETLQQMHRILTTRQAARGLLALGEYFQRLRALSSSWAARQREPT

>AtTGA5

MGDTSPRTSVSTDGDTDHNNLMFDEGHLGIGASDSSDRSKSKMDQKTLRRLAQNREAARKSRLRKKAYVQQLENSRLKLTQLEQELQRARQQGVFISSSGDQAHSTAGDGAMAFDVEYRRWQEDKNRQMKELSSAIDSHATDSELRIIVDGVIAHYEELYRIKGNAAKSDVFHLLSGMWKTPAERCFLWLGGFRSSELLKLIASQLEPLTEQQSLDINNLQQSSQQAEDALSQGMDNLQQSLADTLSSGTLGSSSSGNVASYMGQMAMAMGKLGTLEGFIRQADNLRLQTYQQMVRLLTTRQSARALLAVHNYTLRLRALSSLWLARPRE

>AtTGA6

MHAAASDSSDRSKDKLDQKTLRRLAQNREAARKSRLRKKAYVQQLENSRLKLTQLEQELQRARQQGVFISSSGDQAHSTGGNGALAFDAEHSRWLEEKNRQMNELRSALNAHAGDTELRIIVDGVMAHYEELFRIKSNAAKNDVFHLLSGMWKTPAERCFLWLGGFRSSELLKLLANQLEPMTERQVMGINSLQQTSQQAEDALSQGMESLQQSLADTLSSGTLGSSSSDNVASYMGQMAMAMGQLGTLEGFIRQADNLRLQTLQQMLRVLTTRQSARALLAIHDYSSRLRALSSLWLARPRE

>AtTGA7

MMSSSSPTQLASLRDMGIYEPFQQIVGWGNVFKSDINDHSPNTATSSIIQVDPRIDDHNNNIKINYDSSHNQIEAEQPSSNDNQDDDGRIHDKMKRRLAQNREAARKSRLRKKAYVQQLEESRLKLSQLEQELEKVKQQGHLGPSGSINTGIASFEMEYSHWLQEQSRRVSELRTALQSHISDIELKMLVESCLNHYANLFQMKSDAAKADVFYLISGMWRTSTERFFQWIGGFRPSELLNVVMPYLQPLTDQQILEVRNLQQSSQQAEDALSQGIDKLQQSLAESIVIDAVIESTHYPTHMAAAIENLQALEGFVNQADHLRQQTLQQMAKILTTRQSARGLLALGEYLHRLRALSSLWAARPQEPT

>AtTGA9

MQGHHQNHHQHLSSSSATSSHGNFMNKDGYDIGEIDPSLFLYLDGQGHHDPPSTAPSPLHHHHTTQNLAMRPPTSTLNIFPSQPMHIEPPPSSTHNTDNTRLVPAAQPSGSTRPASDPSMDLTNHSQFHQPPQGSKSIKKEGNRKGLASSDHDIPKSSDPKTLRRLAQNREAARKSRLRKKAYVQQLESCRIKLTQLEQEIQRARSQGVFFGGSLIGGDQQQGGLPIGPGNISSEAAVFDMEYARWLEEQQRLLNELRVATQEHLSENELRMFVDTCLAHYDHLINLKAMVAKTDVFHLISGAWKTPAERCFLWMGGFRPSEIIKVIVNQIEPLTEQQIVGICGLQQSTQEAEEALSQGLEALNQSLSDSIVSDSLPPASAPLPPHLSNFMSHMSLALNKLSALEGFVLQADNLRHQTIHRLNQLLTTRQEARCLLAVAEYFHRLQALSSLWLARPRQDG

>AtTGA10

MQGHHQNHHQHLSSSSATSSHGNFMNKDGYDIGEIDPSLFLYLDGQGHHDPPSTAPSPLHHHHTTQNLAMRPPTSTLNIFPSQPMHIEPPPSSTHNKEGNRKGLASSDHDIPKSSDPKTLRRLAQNREAARKSRLRKKAYVQQLESCRIKLTQLEQEIQRARSQGVFFGGSLIGGDQQQGGLPIGPGNISSAEAAVFDMEYARWLEEQQRLLNELRVATQEHLSENELRMFVDTCLAHYDHLINLKAMVAKTDVFHLISGAWKTPAERCFLWMGGFRPSEIIKVIVNQIEPLTEQQIVGICGLQQSTQEAEEALSQGLEALNQSLSDSIVSDSLPPASAPLPPHLSNFMSHMSLALNKLSALEGFVLQADNLRHQTIHRLNQLLTTRQEARCLLAVAEYFHRLQALSSLWLARPRQDG
